# Optimum strata boundaries and sample sizes in health surveys using auxiliary variables

**DOI:** 10.1371/journal.pone.0194787

**Published:** 2018-04-05

**Authors:** Karuna Garan Reddy, Mohammad G. M. Khan, Sabiha Khan

**Affiliations:** 1 Research Office, Office of Deputy Vice Chancellor (Research, Innovation & International), The University of the South Pacific, Suva, Fiji; 2 School of Computing, Information and Mathematical Sciences, The University of the South Pacific, Suva, Fiji; 3 School of Public Health and Primary Care, Fiji National University, Suva, Fiji; Indraprastha Institute of Information Technology, INDIA

## Abstract

Using convenient stratification criteria such as geographical regions or other natural conditions like age, gender, etc., is not beneficial in order to maximize the precision of the estimates of variables of interest. Thus, one has to look for an efficient stratification design to divide the whole population into homogeneous strata that achieves higher precision in the estimation. In this paper, a procedure for determining Optimum Stratum Boundaries (OSB) and Optimum Sample Sizes (OSS) for each stratum of a variable of interest in health surveys is developed. The determination of OSB and OSS based on the study variable is not feasible in practice since the study variable is not available prior to the survey. Since many variables in health surveys are generally skewed, the proposed technique considers the readily-available auxiliary variables to determine the OSB and OSS. This stratification problem is formulated into a Mathematical Programming Problem (MPP) that seeks minimization of the variance of the estimated population parameter under Neyman allocation. It is then solved for the OSB by using a dynamic programming (DP) technique. A numerical example with a real data set of a population, aiming to estimate the Haemoglobin content in women in a national Iron Deficiency Anaemia survey, is presented to illustrate the procedure developed in this paper. Upon comparisons with other methods available in literature, results reveal that the proposed approach yields a substantial gain in efficiency over the other methods. A simulation study also reveals similar results.

## Introduction

Stratified random sampling is an important sampling technique utilized in estimating the prevalence of diseases such as diabetes, anaemia, obesity hypertension, and smoking. In stratified sampling, the sampling-frame is divided into a number (say, *L*) of non-overlapping groups or strata in such a way that the strata constructed are internally homogeneous with respect to the variable (or main variable) under study, because that maximizes the precision of the estimator of the parameter of interest concerning the study variable, e.g. its mean [1]. An advantage of stratified sampling design is that when a stratum is homogeneous, the measurements of the study variable (*y*) vary little from each other and the precise estimate of *y* can be obtained from a small sample in that stratum. Thus, combining these estimates from all *L* strata, the design produces a gain in the precision of estimate of the variable in the whole population [1]. However, in most practical situations, especially in health surveys, it is difficult to construct such optimum strata, and hence, more often the health surveyors stratify the population in most convenient manners such as the use of geographical regions (e.g. North, Central, South, etc.), administrative regions (e.g. provinces, districts, etc.) or other natural criteria (e.g. urban-rural, sex, age etc.). Moreover, the stratification by convenience manner is not always a reasonable criterion as the strata so obtained may not be internally homogeneous with respect to a variable of interest. Thus, one has to look for the Optimum Stratum Boundaries (OSB) that maximizes the precision of the estimators.

The problem of determining OSB for a variable, when its frequency distribution is known, is well-known in the sampling literature. The basic consideration involved in determining OSB is that the strata should be as internally homogenous as possible, that is, in order to achieve maximum precision, the stratum variances should be as small as possible [1, 2]. When a single variable is of interest and the stratification is made based on this study variable, then an ideal situation is that the distribution of the study variable is known and the OSB can be determined by cutting the range of its distribution at suitable points. This problem of determining the OSB, when both the estimation and stratification variables are the same, was first discussed by Dalenius [3]. He presented a set of minimal equations which are usually difficult to solve for OSB because of their implicit nature. Hence, subsequently the attempts for determining approximately optimum stratum boundaries have been made by several authors [4–9].

Many authors have also attempted to determine the global OSB. For example, Unnithan [10] proposed an iterative method that requires a suitable initial solution. For a skewed population where a certainty stratum (some specific units are included in the sample where extremely large units are isolated so that they do not influence sampling variability) is necessary. Lavallée [11] proposed an algorithm to construct stratum boundaries for a power allocated (applying an exponential value *q*, where 0 < *q* < 1, to the stratum population value under Neyman Allocation to allow for a sufficient spread of the sample allocation) stratified sample. Later on, Hidiroglou [12] presented a more general form of the algorithm. After reviewing Lavallée and Hidiroglou’s algorithm, a modified algorithm that incorporated the different relationships between the stratification and study variables was proposed [13, 14].

There are several other algorithms available in the literature, for example, Niemiro [15] proposed a random search method and the simplex method [16] was used to present a new method of stratification [17]. Later on, Kozak [18] presented a modified random search algorithm. Gunning [19] proposed an alternative method to approximate stratification based on a geometric progression. This approach was compared with three other methods [8, 11, 20] which confirmed that the geometric progression method is more efficient [21]. The usefulness of Gunning and Horgan’s geometric progression method was studied and it revealed that the geometric progression approach is less efficient than Lavallée and Hidiroglou’s algorithm [22, 23].

Another kind of stratification method that has been proposed in the literature is due to Khan et. al. [24–30]. When the distributions of the study variables were known, they formulated the problems of determining OSB as optimization problems, which are solved by developing computational techniques Dynamic Programming (DP). The DP technique was first proposed by Bühler & Deutler [31], which was also used for determining the OSB which would divide the population domain of two stratification variables into distinct subsets such that the precision of the variables of interest is maximized [11, 32].

Numerous research have also been undertaken whereby auxiliary variable(s), which can be historical data, are used to improve the precision of the estimates of study variable *y*. When the frequency distribution of the auxiliary variable, *x*, is known, several approximation methods of determining OSB using the auxiliary variable(s) have been suggested and discussed by many authors [1, 9, 33–43].

In this paper, a procedure for determining the OSB and sample size for each stratum of a variable of interest in health surveys is developed. The determination of the OSB and sample sizes, based directly on the survey variable (*y*), is not feasible in practice since the it is unavailable prior to conducting the survey. Hence, optimum stratification is made based on multiple auxiliary variables (*x*_1_, *x*_2_, …, *x*_*p*_) that are readily available in health surveys. It shall be assumed that the population values of the study variable *y* are available as realizations of a stochastic background variable or at least can be realized as proxy values of *y* from previous or other recent surveys and *y* holds a regression model with the auxiliary variable(s) [2, 14, 30, 44–46]. Moreover, often *y* is highly correlated with *x* such that the regression of *y* upon *x* has homoscedastic errors. In situations like this, stratification can be achieved using the auxiliary variable(s). The application of the proposed methodology will be demonstrated with empirical investigations using real and simulated datasets. This proposed research deals with the problem of stratification for a study variable using the many auxiliary variables that are found in a multivariate survey. In health surveys, these auxiliary variables normally characterize positively skewed distributions that are families of the Gaussian distribution such as Weibull, Gamma, Log-normal, etc. Thus, this research investigates if the proposed parametric-based mathematical programming approach for determining the OSB yields a gain in efficiency over other methods that are well-known in literature. This research also tries to find out if the proposed method works for skewed distributions such as the Weibull or Gamma when both linear and nonlinear regression models are used in the MPP formulation of the stratification problem.

The problem of determining OSB is redefined as the problem of determining Optimum Strata Widths (OSW) and is formulated as a Mathematical Programming Problem (MPP) that seeks minimization of the variance of the estimated population parameter. Since the formulated MPP can be viewed as a multistage decision problem, it is solved using a DP technique. These OSB are then used to compute the sample size for each stratum under Neyman allocation. A numerical example with a real data set of skewed population, where the auxiliary variables follow Weibull distributions, is presented to illustrate the proposed procedure. The results are compared with the Dalenius & Hodges’ cum $\sqrt{f}$ method [20], Gunning & Horgan’s geometric method [19] and Lavallée & Hidiroglou’s method [11].

## The general formulation of the problem of OSB as an MPP

Let the population be stratified into a fixed *L* strata based on *p* auxiliary variables, *x*_1_, *x*_2_, …, *x*_*p*_, and the estimation of the mean of study variable *y* is of interest. If a simple random sample of size *n*_*h*_ is to be drawn from *h*^*th*^ stratum with sample mean ${\bar{y}}_{h};\left(h=1,2,\ldots ,L\right)$, then an unbiased stratified sample mean, ${\bar{y}}_{st}$, is given by

$$\begin{array}{c} {\bar{y}}_{st}=\sum_{h=1}^{L}W_{h}{\bar{y}}_{h}, \end{array}$$ (1)

where *W*_*h*_ = *N*_*h*_/*N* is the proportion of the population contained in the *h*^*th*^ stratum for the study variable *y*, where *N* is the total number of units in the population and is assumed to be known while *N*_*h*_ is the total unknown number of units in each stratum. Then the variance of ${\bar{y}}_{st}$ is given by

$$\begin{array}{c} V\left({\bar{y}}_{st}\right)=\sum_{h=1}^{L}\left(\frac{1}{n_{h}}-\frac{1}{N_{h}}\right)W_{h}^{2}\sigma_{h}^{2}. \end{array}$$ (2)

The finite population correction factors in (2) could be ignored [8, 9, 20, 36]. Thus, under the Neyman allocation [47], that is,

$$\begin{array}{c} n_{h}=n\cdot \frac{W_{h}\sigma_{hy}}{\sum_{h=1}^{L}W_{h}\sigma_{hy}}, \end{array}$$ (3)

(2) is given by

$$\begin{array}{c} V\left({\bar{y}}_{st}\right)=\frac{{\left(\sum_{h=1}^{L}W_{h}\sigma_{hy}\right)}^{2}}{n}, \end{array}$$ (4)

where *σ*_*hy*_ is the stratum standard deviation of *y* in *h*^*th*^ stratum; *h* = 1, 2, …, *L* and *n* is the preassigned total sample size.

Consider that the study variable has the regression model of the form:

$$\begin{array}{c} y=\lambda (x_{1},x_{2},\ldots ,x_{p})+\epsilon , \end{array}$$ (5)

where *λ*(*x*_1_, *x*_2_, …, *x*_*p*_) is a linear (or nonlinear) function of *x*_*i*_;(*i* = 1, 2, …, *p*) and *ϵ* is an error term such that *E*(*ϵ*|*x*_1_, *x*_2_, …, *x*_*p*_) = 0 and *V*(*ϵ*|*x*_1_, *x*_2_, …, *x*_*p*_) = *ψ*(*x*_1_, *x*_2_, …, *x*_*p*_) > 0 for all *x*_*i*_. The parameters in *λ* are assumed to be known from a recent survey.

Assuming that *λ* and *ϵ* are uncorrelated [4], it follows that

$$\begin{array}{c} \sigma_{hy}^{2}=\sigma_{h\lambda (x_{1},x_{2},\ldots ,x_{p})}^{2}+\sigma_{h\epsilon }^{2}, \end{array}$$ (6)

where $\sigma_{h\lambda (x_{1},x_{2},\ldots ,x_{p})}^{2}$ denotes the variance of *λ*(*x*_1_, *x*_2_, …, *x*_*p*_) in the *h*^*th*^ stratum and $\sigma_{h\epsilon }^{2}$ is the variance of *ϵ* in the *h*^*th*^ stratum. Eq (6) assumes homoscedasticity, i.e., homogeneity of the variance of *ϵ* over the distribution of the predictors *x*_*i*_(*i* = 1, 2, …, *p*), given the stratum *h*.

Let *f*(*x*_*i*_) be the estimated frequency functions of the auxiliary variables, *x*_*i*_(*i* = 1, 2, …, *p*), that are used for the stratification of the main variable. If the population mean of the study variable *y* is to be estimated over a range (*a*, *b*) under the allocation (3), then the problem of determining the strata boundaries of *y* is to cut up the range, (*a*, *b*) at (*L* − 1) intermediate points *a* = *y*_0_ ≤ *y*_1_ ≤ *y*_2_ ≤, …, ≤ *y*_*L*−1_ ≤ *y*_*L*_ = *b* such that (4) is minimum. Since the study variable is not available at the design stage, the range (*a*, *b*) could either be the compromise range derived from all the auxiliary variables or an estimated range that best explains the study variable, possibly chosen from previous surveys.

For a fixed sample size *n*, minimizing the expression of the right hand side of (4) is equivalent to minimizing $\sum_{h=1}^{L}W_{h}\sigma_{hy}$. Thus, from (6), the following is minimized:

$$\begin{array}{ccc} \sum_{h=1}^{L}W_{h}\sigma_{hy} & = & \sum_{h=1}^{L}\sqrt{W_{h}^{2}\sigma_{h\lambda (x_{1},x_{2},\ldots ,x_{p})}^{2}+W_{h}^{2}\sigma_{h\epsilon }^{2}} \end{array}$$ (7)

If *f*(*x*_*i*_) are known and integrable frequency functions of the auxiliary variables, then for the given *λ*(*x*_1_, *x*_2_, …, *x*_*p*_), the first term inside the square root function in (7) can be expressed as the functions of the boundary points (*y*_*h*−1_, *y*_*h*_) by finding the stratum weight *W*_*hx*_*i*__, mean *μ*_*hx*_*i*__ and variance $\sigma_{hx_{i}}^{2}$ of *i*th auxiliary variable *x*_*i*_ using the following expressions:

$$\begin{array}{ccc} W_{hx_{i}} & = & \int_{y_{h-1}}^{y_{h}}f\left(x_{i}\right)dx_{i} \end{array}$$ (8)

$$\begin{array}{ccc} \mu_{hx_{i}} & = & \frac{1}{W_{hx_{i}}}\int_{y_{h-1}}^{y_{h}}x_{i}f\left(x_{i}\right)dx_{i} \end{array}$$ (9)

$$\begin{array}{ccc} \sigma_{hx_{i}}^{2} & = & \frac{1}{W_{hx_{i}}}\int_{y_{h-1}}^{y_{h}}x_{i}^{2}f\left(x_{i}\right)dx_{i}-\mu_{hx_{i}}^{2} \end{array}$$ (10)

where *i* = 1, 2, …, *p*.

The quantities computed by Eqs (8)–(10) may be different for different auxiliary variables since it depends on their best-fit frequency distributions, for example, Weibull, Gamma, or any other skewed distribution. If two or more auxiliary variables are characterized by the same distribution, the quantities in (8)–(10) may still be different because their estimated parameters would certainly be different.

Note that the second term in (7) are also obtained as a function of boundary points using the frequency function of the regression error. Thus, the objective function (7) could be expressed as a function of boundary points (*y*_*h*−1_, *y*_*h*_) only: $\phi_{h}\left(y_{h},y_{h-1}\right)=\sqrt{W_{h}^{2}\sigma_{h\lambda }^{2}+W_{h}^{2}\sigma_{h\epsilon }^{2}}$. Then, the problem of determination of OSB can be expressed as the following optimization problem to determine *y*_1_, *y*_2_, …, *y*_*L*_.

$$\begin{array}{ccc} & \text{Minimize}\;\;\;\sum_{h=1}^{L}\phi_{h}\left(y_{h},y_{h-1}\right) & \\ & \text{subject to}\;\;\;a=y_{0}\leq y_{1}\leq y_{2}\leq ,\ldots ,\leq y_{L}=b \end{array}$$ (11)

We further define *l*_*h*_ = *y*_*h*_ − *y*_*h*−1_;*h* = 1, 2, …, *L*, where *l*_*h*_ ≥ 0 denotes the range or width of the *h*^*th*^ stratum. From this, the range of the distribution of *y*, *d* = *b* − *a*, can be expressed as a function of the stratum width.

$$\begin{array}{c} \sum_{h=1}^{L}l_{h}=\sum_{h=1}^{L}\left(y_{h}-y_{h-1}\right)=b-a=y_{L}-y_{0}=d \end{array}$$ (12)

The *h*^*th*^ stratification point *y*_*h*_; *h* = 1, 2, …, *L* is then expressed as

$$\begin{array}{c} y_{h}=y_{0}+\sum_{i=1}^{h}l_{i}=y_{h-1}+l_{h} \end{array}$$ (13)

Adding (12) as a constraint, the problem (11) can be treated as an equivalent problem of determining optimum strata widths (OSW), *l*_1_, *l*_2_, …, *l*_*L*_, and is expressed as:

$$\begin{array}{ccc} & \text{Minimize} & \sum_{h=1}^{L}\phi_{h}\left(l_{h},y_{h-1}\right),\\ & \text{subject to} & \sum_{h=1}^{L}l_{h}=d,\\ & \text{and} & \;\;\;\;l_{h}\geq 0;h=1,2,\ldots ,L. \end{array}$$ (14)

Note that if *y*_0_ is known, the first term, *ϕ*_1_(*l*_1_, *y*_0_), in the objective function of the MPP (14) is a function of *l*_1_ alone. Once *l*_1_ is known, the second term *ϕ*_2_(*l*_2_, *y*_1_) will become a function of *l*_2_ alone and so on. Due to the special nature of functions, the MPP (14) may be treated as a function of *l*_*h*_ alone and is expressed as:

$$\begin{array}{ccc} & \text{Minimize} & \;\;\;\sum_{h=1}^{L}\phi_{h}\left(l_{h}\right),\\ & \text{subject to} & \;\;\;\;\sum_{h=1}^{L}l_{h}=d,\\ & \text{and} & \;\;\;\;l_{h}\geq 0;\;\;h=1,2,\ldots ,L. \end{array}$$ (15)

In real-world situations, the study variable is unknown at the design stage, hence, readily-available auxiliary variables can be used to create OSB. The proposed technique carries out optimization through the MPP (15) on the compromise range (*d*) derived from all auxiliary variables. The technique also assumes that the parameters of the regression model in (15) are known from a recent survey. The best-fit distributions of the auxiliary variables, *x*_*i*_, are used in the formulation of MPP (15).

## The solution procedure using dynamic programming technique

The problem (15) is a multistage decision problem in which the objective function and the constraint are separable functions of *l*_*h*_, which allows us to use a DP technique [28]. Dynamic programming determines the optimum solution of a multi-variable problem by decomposing it into stages, each stage compromising a single variable subproblem. A DP model is basically a recursive equation based on Bellman’s principle of optimality [48]. This recursive equation links the different stages of the problem in a manner which guarantees that each stage’s optimal feasible solution is also optimal and feasible for the entire problem [49].

Consider the following subproblem of (15) for first *k*(< *L*) strata:

$$\begin{array}{ccc} & \text{Minimize} & \;\;\;\;\sum_{h=1}^{k}\phi_{h}\left(l_{h}\right),\\ & \text{subject to} & \;\;\;\;\;\sum_{h=1}^{k}l_{h}=d_{k},\\ & \text{and} & \;\;\;\;\;l_{h}\geq 0;\;\;h=1,2,\ldots ,k. \end{array}$$ (16)

where *d*_*k*_ < *d* is the total width available for division into *k* strata or the state value at stage *k*. Note that *d*_*k*_ = *d* for *k* = *L* and the transformation functions are given by

$$\begin{array}{ccc} d_{k}\;\; & = & \;\;l_{1}+l_{2}+\ldots +l_{k},\;\;\text{and}\\ d_{k-1}\;\; & = & \;\;l_{1}+l_{2}+\ldots +l_{k-1}=d_{k}-l_{k} \end{array}$$

Let Φ_*k*_(*d*_*k*_) denote the minimum value of the objective function of (16), that is,

$$\begin{array}{ccc} \Phi_{k}\left(d_{k}\right) & = & \text{min}[\sum_{h=1}^{k}\phi_{h}\left(l_{h}\right)|\sum_{h=1}^{k}l_{h}=d_{k},\text{and}\\ & & \;\;\;l_{h}\;\geq 0;\;\;h=1,2,\ldots ,k\;\text{and}\;1\leq k\leq L] \end{array}$$

With the above definition of Φ_*k*_(*d*_*k*_), the MPP (15) is equivalent to finding Φ_*L*_(*d*) recursively by finding Φ_*k*_(*d*_*k*_) for *k* = 1, 2, …, *L* and 0 ≤ *d*_*k*_ ≤ *d*. We can write:

$$\begin{array}{ccc} \Phi_{k}\left(d_{k}\right) & = & \text{min}[\phi_{k}\left(l_{k}\right)+\sum_{h=1}^{k-1}\phi_{h}\left(l_{h}\right)|\sum_{h=1}^{k-1}l_{h}=d_{k}-l_{k},\\ & & \;\;\;\;\;and\;l_{h}\;\geq \;0;\;h=1,2,\ldots ,k] \end{array}$$

For a fixed value of *l*_*k*_; 0 ≤ *l*_*k*_ ≤ *d*_*k*_,

$$\begin{array}{ccc} \Phi_{k}\left(d_{k}\right) & = & \phi_{k}\left(l_{k}\right)+\text{min}\left[\sum_{h=1}^{k-1}\phi_{h}\left(l_{h}\right)\right|\sum_{h=1}^{k-1}l_{h}=d_{k}-l_{k},\\ & & \text{and}\;l_{h}\;\geq 0;\;h=1,2,\ldots k-1;\;1\leq k\leq L] \end{array}$$

Using the Bellman’s principle of optimality, we write a forward recursive equation of the DP technique for *k* ≥ 2 as:

Φk(dk)=min0≤lk≤dk[ϕk(lk)+Φk-1(dk-lk)] (17)

For the first stage, that is, for *k* = 1:

$$\begin{array}{c} \Phi_{1}\left(d_{1}\right)=\phi_{1}\left(d_{1}\right)\;⟹\;\;l_{1}^{*}=d_{1}, \end{array}$$ (18)

where $l_{1}^{*}=d_{1}$ is the optimum width of the first stratum. The relations (17) and (18) are solved in a forward manner first for *k* = 1, 2, …, *L* to determine the optimum subproblem objective and then solved in a backward manner second to determine the OSB.

The application of the above solution procedure is summarized in Appendix A in order to determine the OSB for MPP (15).

## Determination of optimum sample size

When OSB (*y*_*h*_, *y*_*h*−1_) are determined as discussed in Sections 2-3, the optimum sample size *n*_*h*_;*h* = 1, 2, …, *L* that minimizes the variance of the estimate can easily be computed.

If the study variable holds the regression model (5) with the auxiliary variables across the strata, using (2) and (7), the sample size *n*_*h*_ are obtained for a fixed total sample of size *n* under Neyman allocation [47] for *h* = 1, 2, …, *L* and given as follows:

$$\begin{array}{c} n_{h}=n\;\frac{W_{h}\sqrt{\sigma_{h\lambda (x_{1},x_{2},\ldots ,x_{p})}^{2}+\sigma_{h\epsilon }^{2}}}{\sum_{h=1}^{L}W_{h}\sqrt{\sigma_{h\lambda (x_{1},x_{2},\ldots ,x_{p})}^{2}+\sigma_{h\epsilon }^{2}}} \end{array}$$ (19)

where *W*_*h*_, $\sigma_{h\lambda (x_{1},x_{2},\ldots ,x_{p})}^{2}$ and $\sigma_{h\epsilon }^{2}$ are derived in terms of the optimum boundary points (*y*_*h*_, *y*_*h*−1_).

It is also worth noting that the OSB (*y*_*h*_, *y*_*h*−1_) through the MPP (15) are so obtained that *n*_*h*_ must satisfy the restrictions:

$$\begin{array}{c} 1\leq n_{h}\leq N_{h}, \end{array}$$ (20)

where *N*_*h*_ = *NW*_*h*_. The restriction 1 ≤ *n*_*h*_ is added to the formulation so that the *h*^*th*^ stratum must form with at least a unit and the restriction *n*_*h*_ ≤ *N*_*h*_ is added to avoid the over sampling.

## Determination of optimum number of strata

This is one of the first issues that need to be considered in an optimal stratification design, however, it can be dependent on the OSB and the allocation of sample units among the strata. The goal of stratification is to make all strata as homogenous as possible, which implies that the more the number of strata, the more the homogeneity within a stratum. This results in a reduction in the total variance of ${\bar{y}}_{st}$, that is, $Var({\bar{y}}_{st})$. However, an increase in the number of strata may involve extra cost and resources in planning and drawing the samples.

Problem of determining optimum number of strata was first discussed by Dalenius [3] who postulated that uniformly distributed variable, $Var({\bar{y}}_{st})$ is inversely proportional to *L*^2^. Later, Cochran [50] investigated the effect of the number of strata on $Var({\bar{y}}_{st})$ for some skewed distributed populations with Neyman allocation. He confirmed that this relationship holds for skewed distribution and the rate of reduction in $Var({\bar{y}}_{st})$ is independent of skewness of the population. The results indicated that only a little reduction in variance is to be expected beyond *L* = 6 unless the correlation between the auxiliary information and the survey population is greater than 0.95.

To apply the above idea to the current situation of the optimal number of strata, assume that fpc is negligible and consider the distribution of the data to be approximately uniform, as done by Cochran [1]. Then the range of the distribution of values of *y* [*a*, *b*] is *d* = *b* − *a*, and hence the variance of the distribution is *S*^2^ = *d*^2^/12. The variance of the sample mean for a simple random sample of size *n* can therefore be calculated as:

$$\begin{array}{ccc} V(\bar{y}) & = & \frac{S^{2}}{n}=\frac{S^{2}}{12n} \end{array}$$ (21)

Thus, if a simple case of creating *L* strata of equal size is considered, stratum variance would then be calculated as *S*^2^ = *d*^2^/12*L*^2^. It follows from *W*_*h*_ = 1/*L* and Eq (4),

$$\begin{array}{ccc} V({\bar{y}}_{st}) & = & \frac{1}{n}\sum_{h=1}^{L}{\left(\frac{1}{L}\frac{d}{\sqrt{12}L}\right)}^{2}=\frac{1}{n}{\left(\frac{d}{\sqrt{12}L}\right)}^{2}\\ & = & \frac{d^{2}}{12nL^{2}}=\frac{V(\bar{y})}{L^{2}} \end{array}$$ (22)

This reveals that variance of the sample mean is inversely proportional to the square of the number of strata, *L*. This however, does not consider the relationship between the auxiliary variables and the study population. It can be extended by considering a linear relationship given in Eq (5). As suggested by Cochran [50], in this case, using (5), it can be shown that

$$\begin{array}{c} V\left({\bar{y}}_{st}\right)=\frac{1}{n}\left(\frac{\sigma_{\lambda }^{2}}{L^{2}}+\sigma_{\epsilon }^{2}\right) \end{array}$$ (23)

**Fig 1:**
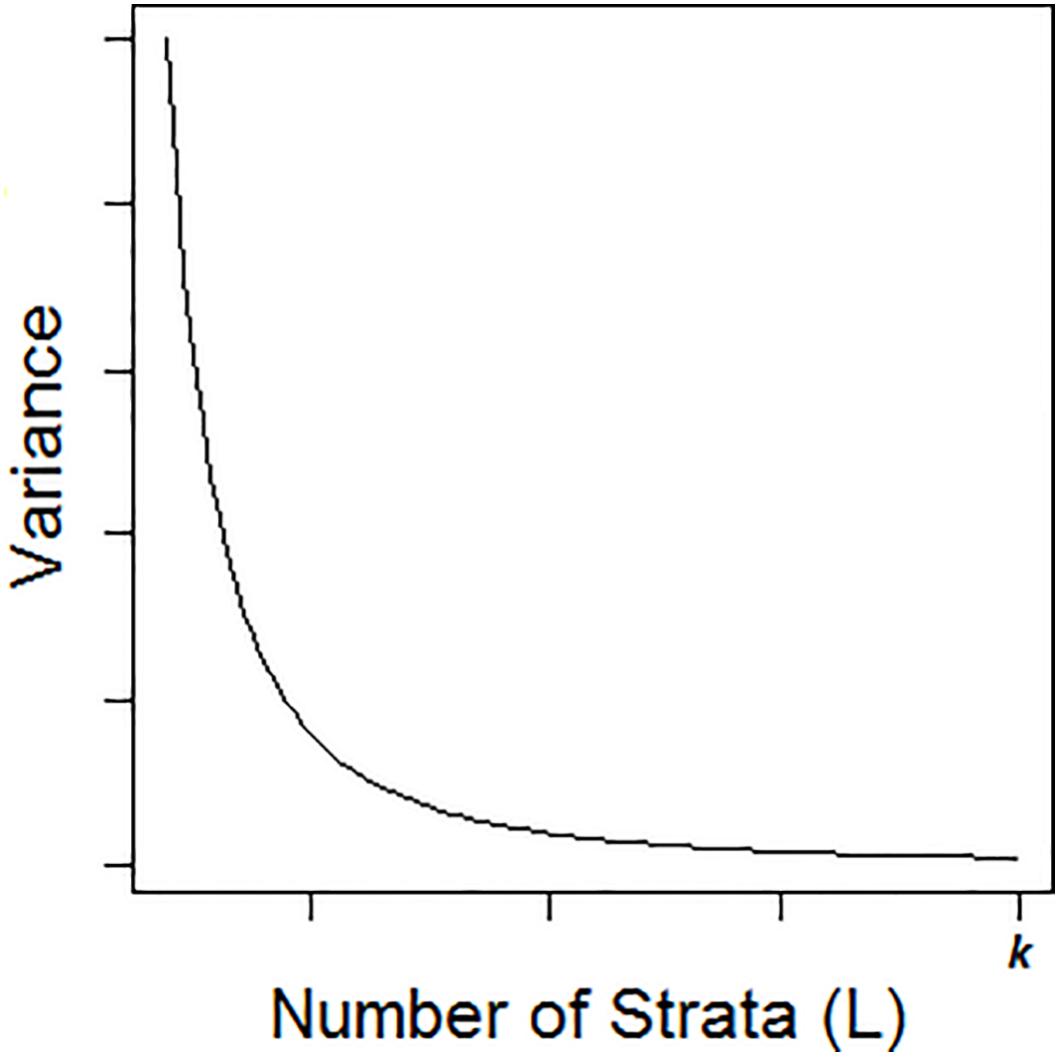
Plot of $V({\bar{y}}_{st})$ against *L*.

This again shows that the variance is inversely related to the square of the number of strata. Applying (23), one can empirically study the effect of increasing the number of strata. To complete this analysis, a cost function that shows the relation of cost with *L*, for planning and executing a survey, is required. However, whatever the form of cost function, [1] showed that the increase in *L* beyond 6 will seldom be profitable. Thus, if the extra cost involved in planning and executing the survey, which is incurred due to an increase in the number of strata is not of much importance, a reasonable approach to determining the optimum number of strata may be discussed as follows:

Compute $V({\bar{y}}_{st})$ for *L* = 1, 2, ‥, *k*, where *k* is a possible value of the candidate *L*. Now $V({\bar{y}}_{st})$ decreases as *L* increases and $V({\bar{y}}_{st})$ is minimum when *L* = *k*. Therefore, a surveyor may choose the optimum number of strata at the point where an increase in *L* is not useful as it gives only a small decrease in $V({\bar{y}}_{st})$. The approach is illustrated in Fig 1, which is a hypothetical plot of $V({\bar{y}}_{st})$ against *L*. One can choose the desired number of strata as the point at which the “elbow” in the curve becomes apparent. Clearly, this requires judgment on the part of the surveyors.

## Construction of OSB with Weibull auxiliary variables

The Weibull distribution is a two or three-parameter family of continuous probability distributions. Because of its versatility in the fitting of a variety of distributions, it is one of the most widely used distributions in applied statistics, especially in survival analysis, mortality or failure analysis, reliability, engineering to model manufacturing and delivery times, in extreme value theory and weather forecasting. Due to its moderately skewed profile, it also characterizes well a wide range of health data, including health monitoring data, epidemiological data such as episode durations of depression and gene expressions data [51–54].

If all the auxiliary variables, *x*_*i*_;*i* = 1, 2, …, *p*, approximately follow Weibull distribution on the interval [*x*_*i*,0_, *x*_*i*, *L*_], its three-parameter probability density function with a state space *x*_*i*_ ≥ 0 is given by:

$$\begin{array}{c} f\left(x_{i};r_{i},\theta_{i},\gamma_{i}\right)=\frac{r_{i}}{\theta_{i}}{\left(\frac{x_{i}-\gamma_{i}}{\theta_{i}}\right)}^{r_{i}-1}e^{-{\left(\frac{x_{i}-\gamma_{i}}{\theta_{i}}\right)}^{r_{i}}},\;\;x_{i}\geq 0 \end{array}$$ (24)

where *r*_*i*_ > 0 is the shape parameter, *θ*_*i*_ > 0 is the scale parameter and *γ*_*i*_ is the location parameter of the distribution of *i*^*th*^ auxiliary variable.

The shape parameter gives the Weibull distribution its flexibility. By changing the value of the shape parameter, the distribution can model a wide variety of data that follows the Exponential distribution, the Rayleigh distribution, the Normal distribution or even the approximate Log-normal distribution.

### Scaling the auxiliary variables

While stratifying the study variable based on multiple auxiliary variables, the raw data in the form of different auxiliary variables are generally of different scales (eg., kg, mg, dollar, etc.). The values of one variable may be less or more spread out than other variables. With the auxiliary variables exhibiting different distributions, the range of data, minimum and maximum values for these auxiliary variables will certainly be different from each other. Hence, this may affect the convergence of the MPP (15) and hence its ability to determine the OSB accurately. A way to encounter this problem is to standardize each variable by subtracting its mean and dividing by its standard deviation.

Another method, which this paper uses, is a simple scaling procedure whereby every variable is divided by its maximum value. While maintaining the original distribution of the auxiliary variables, this scaling procedure results in the auxiliary variables getting closer to each other, which in turn, helps in reducing the overall range or the search space of the optimal solution. One must note that the solution procedure of dynamic programming technique is generally advisable and feasible for small sets of units (*N* ≤ 20) [55], hence, scaling is a necessary means for faster convergence of an optimal solution. The MPP (15), when solved, provides the OSB of the scaled study variable and the OSB for the original study variable can be obtained by the usual re-scaling procedure.

### Estimating the regression model

To illustrate the estimation of the regression model in formulating the problem of determining OSB as an MPP for a population with more than one auxiliary variable, we use a health survey data on Anameia, which was obtained from the 2004 Fiji National Nutritional Survey conducted by the National Food and Nutrition Centre (Fiji) and funded by AusAID, UNICEF and Government of Fiji. The data included a micronutrient survey where blood samples were drawn from women of childbearing age and measurements were made to record levels of Haemoglobin, Iron and Folate amongst many other variables. Whilst only tabulations are publicly available from http://ghdx.healthdata.org/record/fiji-national-nutrition-survey-2004, data used for the purpose of applying the proposed method is accessible from http://repository.usp.ac.fj/id/eprint/10439 where the main aim is to estimate the Haemoglobin content in Fijian women. The whole data was fully anonymized before making them accessible. The data cannot be de-anonymized because there is no public datasets available to cross-reference.

The data has the following three characteristics for each woman:

1. Level of Haemoglobin
2. Level of Iron
3. Level of Folate

Suppose that a survey on Iron Deficiency Anaemia is to be conducted in a country, where Haemoglobin (*y*) is the variable of interest and is to be stratified. Then, the levels of Iron and Folate collected in this study may be the reasonable choice for the auxiliary variables, *x*_1_ and *x*_2_. In this example, Haemoglobin is available to us but in reality the main variable might not be available prior to the survey. Thus, Haemoglobin will be used purely as an example for numerical illustrations and comparison purposes.

To estimate the Haemoglobin content (*y*) in women, a multiple regression model (given by Eq (4)) was fitted using scaled data for the survey mentioned above. It was observed that the data significantly fitted a linear regression model with Iron and Folate levels (*p* < 0.001)—the estimated parameters for these two predictors were also highly significant (*p* < 0.001).

The coefficient of determination *R*^2^ = *SSR*/*SST*, with an Adjusted R-squared value of 12.54% was found to be one of the highest for the linear model when compared with the model summary of all the other non-linear models available in standard statistical packages. Thus, this model fits the data best and gives us no reason to consider an alternative model. There is a small positive linear relationship between Haemoglobin and Iron (*r* = 0.350, *p* < 2.2*e* − 16), and Haemoglobin and Folate (*r* = 0.161, *p* < 1.31*e* − 05). Therefore, the Haemoglobin content (*y*), Iron level (*x*_1_) and Folate level (*x*_2_) are fairly assumed to follow a linear regression model given in (5):

$$\begin{array}{c} \lambda \left(x_{1},x_{2}\right)=\beta_{0}+\beta_{1}x_{1}+\beta_{2}x_{2} \end{array}$$ (25)

This idea can be applied in the ideal situation where the main variable is not available. The beta weights of the regression model, initial and final values could be taken as guestimates from prior surveys.

### Estimating the distribution of the auxiliary variables

**Fig 2:**
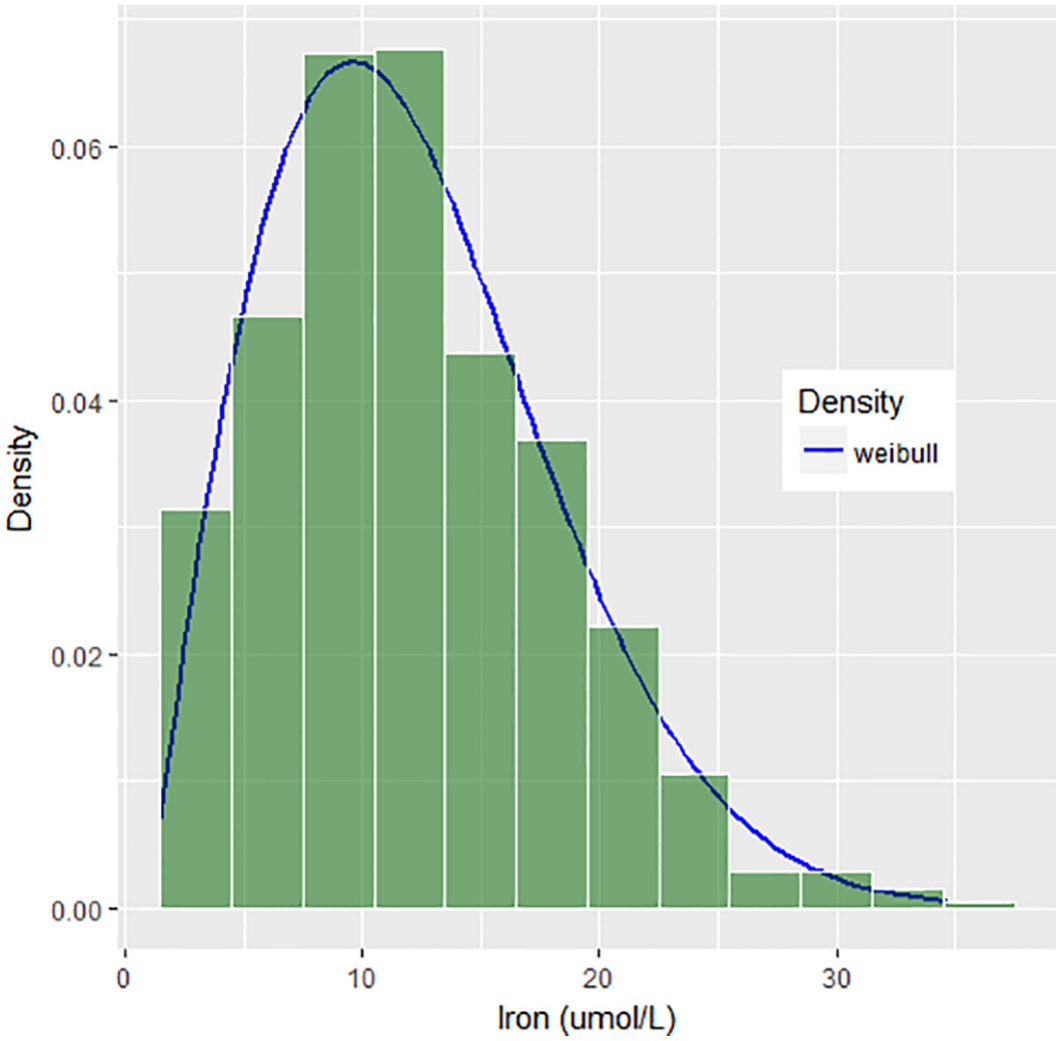
Histogram with density curve for iron.

**Fig 3:**
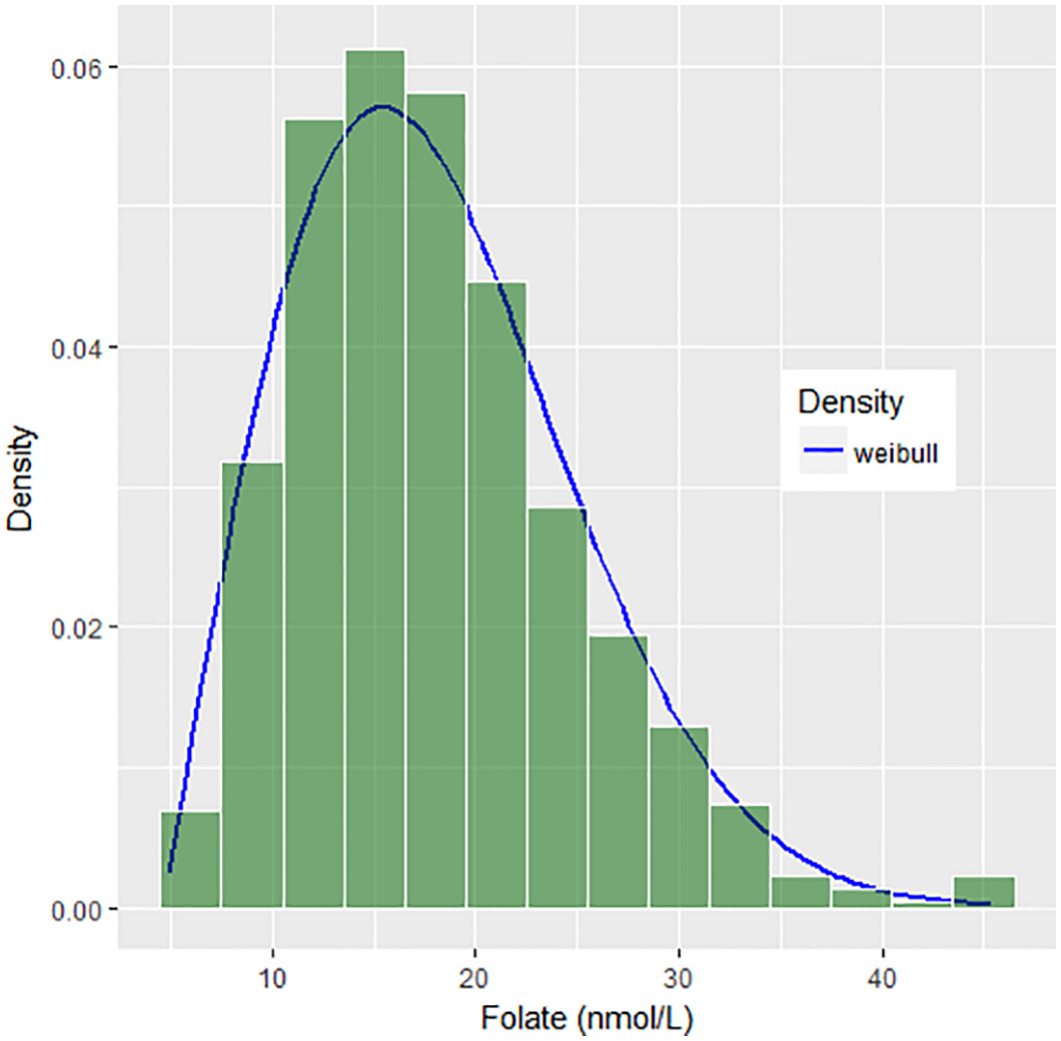
Histogram with density curve for folate.

To determine the distributions of the auxiliary variables, *f*(*x*_1_) and *f*(*x*_2_), relative frequency histograms for Iron and Folate are constructed. The two histograms presented in Figs 2 and 3 reveal that the distributions of both auxiliary variables are right-skewed and match 3P Weibull distribution with different parameters.

Using the Kolmogorov-Smirnov test for each of the two variables, the maximum difference (*D*) between the observed distribution and the Weibull distribution is found to be non-significant (all *p*-values > 0.05). This also supports the fact that all variables follow 3P Weibull distributions, where parameters are obtained by the maximum likelihood estimate (MLE) method.

### Formulation of the MPP with Weibull distribution

Considering that *y* has a linear regression on *x*_*i*_; (*i* = 1, 2, …, *p*). Then, from (5), the function *λ*(*x*_1_, *x*_2_, …, *x*_*p*_) is of the form:

$$\begin{array}{c} \lambda \left(x_{1},x_{2},\ldots ,x_{p}\right)=\alpha +\sum_{i=1}^{p}\beta_{i}x_{i} \end{array}$$ (26)

Assume that the model in (26) holds for all strata. Then,

$$\begin{array}{c} \sigma_{h\lambda (x_{1},x_{2},\ldots ,x_{p})}^{2}=\sum_{i=1}^{p}\beta_{i}^{2}\sigma_{hx_{i}}^{2} \end{array}$$ (27)

Let all the auxiliary variables, *x*_*i*_, follow 3P Weibull distribution (i.e., *x*_*i*_ ∼ *W*(*r*_*i*_, *θ*_*i*_), *γ*_*i*_) with density function given by (24). By using (8)–(10), the quantities *W*_*hx*_*i*__, *μ*_*hx*_*i*__, and $\sigma_{hx_{i}}^{2}$ can be obtained as a function of boundary points (*y*_*h*−1_, *y*_*h*_). Using the substitution of *y*_*h*_ = *y*_*h*−1_+*l*_*h*_, they are presented as follows:

$$\begin{array}{ccc} W_{hx_{i}} & = & e^{-{\left(\frac{y_{h-1}-\gamma_{i}}{\theta_{i}}\right)}^{r_{i}}}-e^{-{\left(\frac{y_{h-1}+l_{h}-\gamma_{i}}{\theta_{i}}\right)}^{r_{i}}} \end{array}$$ (28)

*μ*_*hλ*_ can be expressed as:

$$\begin{array}{ccc} \mu_{hx_{i}} & = & \frac{\theta_{i}}{W_{hx_{i}}}[\int_{{\left(\frac{y_{h-1}-\gamma_{i}}{\theta_{i}}\right)}^{r_{i}}}^{\infty }\;\;t^{\frac{1}{r_{i}}}\;e^{-t}\;dt\\ & & -\int_{{\left(\frac{y_{h}-\gamma_{i}}{\theta_{i}}\right)}^{r_{i}}}^{\infty }\;\;t^{\frac{1}{r_{i}}}\;e^{-t}\;dt] \end{array}$$ (29)

Let Γ(*r*, *x*) and *Q*(*r*, *s*) denote the upper incomplete gamma function and the regularized incomplete gamma function, respectively, given by

$$\begin{array}{c} \Gamma \left(r,x\right)=\int_{x}^{\infty }t^{r-1}e^{-t}\;dt \end{array}$$ (30)

$$\begin{array}{c} Q\left(r,x\right)=\frac{1}{\Gamma (r)}\int_{x}^{\infty }t^{r-1}e^{-t}\;dt,\;\;\;r,x>0;\;\;\Gamma \left(r\right)\neq 0 \end{array}$$ (31)

Then, using Eqs (28)–(31), *μ*_*hx*_*i*__ can be simplified to be

$$\begin{array}{ccc} \mu_{hx_{i}} & = & \frac{\theta_{i}\Gamma \left(1+\frac{1}{r_{i}}\right)}{W_{hx_{i}}}\{[Q\left(1+\frac{1}{r_{i}},{\left(\frac{y_{h-1}-\gamma_{i}}{\theta_{i}}\right)}^{r_{i}}\right)\\ & & -\;Q(1+\frac{1}{r_{i}},{\left(\frac{y_{h-1}+l_{h}-\gamma_{i}}{\theta_{i}}\right)}^{r_{i}})]\} \end{array}$$ (32)

Similarly, the quantity $\sigma_{hx_{i}}^{2}$ is reduced to

$$\begin{array}{ccc} \sigma_{hx_{i}}^{2} & = & \frac{\theta_{i}^{2}\;\Gamma \left(1+\frac{2}{r_{i}}\right)}{W_{hx_{i}}}[Q\left(1+\frac{2}{r_{i}},{\left(\frac{y_{h-1}-\gamma_{i}}{\theta_{i}}\right)}^{r_{i}}\right)\\ & & -\;Q\left(1+\frac{2}{r_{i}},{\left(\frac{y_{h-1}+l_{h}-\gamma_{i}}{\theta_{i}}\right)}^{r_{i}}\right)]-\mu_{hx_{i}}^{2} \end{array}$$ (33)

where *W*_*hx*_*i*__ and and $\mu_{hx_{i}}^{2}$ are given by Eqs (28) and (32) respectively.

Since the auxiliary variables follow Weibull distributions, *W*_*h*_ and $\sigma_{h\lambda }^{2}$ in the first term of (7) are given by (28) and (33) respectively. Thus, for the *i*^*th*^ auxiliary variable, $W_{hx_{i}}^{2}\sigma_{hx_{i}}^{2}$ is

$$\begin{array}{ccc} & = & \frac{\theta_{i}^{2}\;\Gamma \left(1+\frac{2}{r_{i}}\right)}{W_{hx_{i}}}[Q\left(1+\frac{2}{r_{i}},{\left(\frac{y_{h-1}-\gamma_{i}}{\theta_{i}}\right)}^{r_{i}}\right)\\ & & -\;Q(1+\frac{2}{r_{i}},{\left(\frac{y_{h-1}+l_{h}-\gamma_{i}}{\theta_{i}}\right)}^{r_{i}})]\\ & & -[\frac{\theta_{i}\Gamma \left(1+\frac{1}{r_{i}}\right)}{W_{hx_{i}}}[Q\left(1+\frac{1}{r_{i}},{\left(\frac{y_{h-1}-\gamma_{i}}{\theta_{i}}\right)}^{r_{i}}\right)\\ & & -\;{Q(1+\frac{1}{r_{i}},{\left(\frac{y_{h-1}+l_{h}-\gamma_{i}}{\theta_{i}}\right)}^{r_{i}})]]}^{2} \end{array}$$ (34)

Using (34), the formulated MPP given in (15) could be generalised and expressed as the following MPP in order to determine the OSB for the main variable:

$$\begin{array}{ccc} \text{Minimize} & & \sum_{h=1}^{L}\{\text{SQRT}\{\sum_{i=1}^{p}\beta_{i}^{2}\frac{\theta_{i}^{2}\;\Gamma \left(1+\frac{2}{r_{i}}\right)}{W_{hx_{i}}}\\ & & \times \;[Q\left(1+\frac{2}{r_{i}},{\left(\frac{y_{h-1}-\gamma_{i}}{\theta_{i}}\right)}^{r_{i}}\right)\\ & & -\;Q(1+\frac{2}{r_{i}},{\left(\frac{y_{h-1}+l_{h}-\gamma_{i}}{\theta_{i}}\right)}^{r_{i}})]\\ & & -\;[\frac{\theta_{i}\Gamma \left(1+\frac{1}{r_{i}}\right)}{W_{hx_{i}}}[Q\left(1+\frac{1}{r_{i}},{\left(\frac{y_{h-1}-\gamma_{i}}{\theta_{i}}\right)}^{r_{i}}\right)\\ & & -\;{Q(1+\frac{1}{r_{i}},{\left(\frac{y_{h-1}+l_{h}-\gamma_{i}}{\theta_{i}}\right)}^{r_{i}})]]}^{2}\\ & & +\;W_{h}^{2}\sigma_{h\epsilon }^{2}\}\},\\ \text{Subject to} & & \sum_{h=1}^{L}l_{h}=d,\\ \text{and} & & l_{h}\geq 0;\;\;h=1,2,\ldots ,L \end{array}$$ (35)

where *d* in Eq (35) is the estimated or hypothetical range of the main study variable, *β*_*i*_ are the regression coefficients, *θ*_*i*_ and *r*_*i*_ are parameters of the 3P Weibull distributions of *i*^*th*^ auxiliary variable, Γ(⋅) is the upper incomplete gamma function and *Q*(⋅) is the upper regularized incomplete gamma function. Whereas, the term $W_{h}^{2}\sigma_{h\epsilon }^{2}$ can be computed when the distribution of *ϵ* is known. For the current model, since this error term is normally distributed, the distribution is given by:

$$\begin{array}{ccc} f(\epsilon ) & = & \frac{1}{\sqrt{2\pi }}\;\text{exp}\left(-\frac{\epsilon^{2}}{2}\right),\;-\infty <\epsilon <+\infty \end{array}$$ (36)

Then, following from (8)–(10), *W*_*h*_ and *σ*_*hϵ*_ are obtained as:

$$\begin{array}{ccc} W_{h} & = & \frac{erf\left(\frac{y_{h-1}+l_{h}}{\sqrt{2}}\right)-erf\left(\frac{y_{h-1}}{\sqrt{2}}\right)}{2} \end{array}$$ (37)

$$\begin{array}{ccc} \sigma_{h\epsilon }^{2} & = & \{\sqrt{2\pi }[y_{h-1}\;\text{exp}\left(-\frac{y_{h-1}^{2}}{2}\right)erf\left(\frac{y_{h-1}+l_{h}}{\sqrt{2}}\right)\\ & & -\left(y_{h-1}+l_{h}\right)\;\text{exp}\left(-\frac{{\left(y_{h-1}+l_{h}\right)}^{2}}{2}\right)erf\left(\frac{y_{h-1}+l_{h}}{\sqrt{2}}\right)\\ & & -y_{h-1}\;\text{exp}\left(-\frac{y_{h-1}^{2}}{2}\right)erf\left(\frac{y_{h-1}}{\sqrt{2}}\right)\\ & & +\left(y_{h-1}+l_{h}\right)\;\text{exp}\left(-\frac{{\left(y_{h-1}+l_{h}\right)}^{2}}{2}\right)erf\left(\frac{y_{h-1}}{\sqrt{2}}\right)]\\ & & +\pi {\left[erf\left(\frac{y_{h-1}+l_{h}}{\sqrt{2}}\right)-erf\left(\frac{y_{h-1}}{\sqrt{2}}\right)\right]}^{2}\\ & & -2{\left[\text{exp}\left(-\frac{y_{h-1}^{2}}{2}\right)-\text{exp}\left(-\frac{{\left(y_{h-1}+l_{h}\right)}^{2}}{2}\right)\right]}^{2}\}\\ & & ÷\pi {\left[erf\left(\frac{y_{h-1}+l_{h}}{\sqrt{2}}\right)-erf\left(\frac{y_{h-1}}{\sqrt{2}}\right)\right]}^{2} \end{array}$$ (38)

where $erf\left(y_{h}\right)-erf\left(y_{h-1}\right)=\frac{2}{\sqrt{\pi }}\int_{y_{h-1}}^{y_{h}}\text{exp}\left(-u^{2}\right)\;du$ and *h* = 1, 2, …, *L*.

## Numerical illustrations

In this section, numerical results are presented to illustrate the application of the proposed technique to a real and a simulated population. The OSB for the main variable are obtained and presented together with the values of the objective function $\left(\phi_{h}\left(l_{h}\right)=\sum_{h=1}^{L}W_{h}\sigma_{h}\right)$ for *L* = 2, 3, …, 6 for different regression models.

### Real data

The real data, as discussed earlier in Section 6.2, has Haemoglobin as the study variable while Iron and Folate are auxiliary variables that follow Weibull distributions with their estimated parameters. Haemoglobin is being used here purely for comparison purposes, in reality, the main variable is not available. Using the recursive Eqs (17) and (18), the MPP (35) with *d* = 10.9 (range of main variable) is solved by executing a C++ computer program developed to implement the proposed DP technique. R codes were also developed for computing the quantities such as the initial value (*x*_0_) of the distribution, regression coefficients (*β*_*i*_), Weibull parameters (*α*, *β*, *γ*), range (*d*) of the distribution, etc. required for determining the OSB using the C++ program. Users can easily stratify a population by executing the C++ program for the given value of *L*, *x*_0_, *d*, *n*, etc. in an open source IDE such as DEV C++. The C++ program and R codes can be made available on request from the authors.

The results for the OSB (*y*_*h*_) along with optimum sample sizes (*n*_*h*_) and the values of the objective function $\left(\sum_{h=1}^{L}W_{h}\sigma_{h}\right)$ are presented in Table 1 for the following regression models:

$$\begin{array}{ccc} \text{Model}\;1:\;Haemoglobin & = & \beta_{0}+\beta_{1}Iron\\ \text{Model}\;2:\;Haemoglobin & = & \beta_{0}+\beta_{1}Folate\\ \text{Model}\;3:\;Haemoglobin & = & \beta_{0}+\beta_{1}Iron+\beta_{2}Folate \end{array}$$ (39)

**Table 1:** Results for real data using 3P Weibull distribution.

	Model 1			Model 2			Model 3		
L	OSB	*n*_*h*_	$\sum_{h=1}^{L}W_{h}\sigma_{h}$	OSB	*n*_*h*_	$\sum_{h=1}^{L}W_{h}\sigma_{h}$	OSB	*n*_*h*_	$\sum_{h=1}^{L}W_{h}\sigma_{h}$
2	11.04	107	0.094	11.15	116	0.024	11.05	107	0.089
		393			384			393	
3	8.86	20		9.30	22		9.16	20	
	12.84	310	0.063	12.93	319	0.017	12.84	310	0.060
		170			159			170	
4	8.34	9		8.47	11		8.34	9	
	10.93	93	0.048	11.07	102	0.013	10.93	93	0.045
	13.8	334		13.88	321		13.8	335	
		64			66			64	
5	7.87	7		7.98	7		7.87	7	
	9.85	35		9.99	40		9.85	35	
	12.04	162	0.038	12.16	170	0.010	12.04	162	0.036
	14.4	252		14.46	242		14.4	253	
		44			41			44	
6	7.56	5		7.66	6		7.57	5	
	9.16	17		9.30	22		9.16	17	
	10.92	80	0.032	11.06	83	0.009	10.92	80	0.030
	12.81	206		12.91	206		12.81	206	
	14.8	169		14.85	163		14.8	168	
		23			21			23	

### Simulated data

A skewed population with two auxiliary variables (*x*_1_ and *x*_2_) and the study variable (*y*), each of size *N* = 5000, were randomly generated using the *R* software. This data had a relatively weak linear relationship between *y* and *x*_1_ (*r* = 0.014, *p* = 0.34), and a weak linear relationship as well between *y* and *x*_2_ (*r* = 0.023, *p* = 0.11). The simulated data was different from the real data in the sense that it had a very low predictive power in its regression models (Adj. *R*^2^ = 0.03%). The ANOVA results from multiple linear regression also indicated a non-statistically significant model fit (*p* = 0.175).

For the simulated data, the OSB (*y*_*h*_) along with optimum sample sizes (*n*_*h*_) and ∑*W*_*h*_ *σ*_*h*_ values are presented in Table 2 for the following regression models:

$$\begin{array}{ccc} \text{Model}\;4:\;y & = & \beta_{0}+\beta_{1}x_{1}\\ \text{Model}\;5:\;y & = & \beta_{0}+\beta_{1}x_{2}\\ \text{Model}\;6:\;y & = & \beta_{0}+\beta_{1}x_{1}+\beta_{2}x_{2} \end{array}$$

**Table 2:** Results for simulated data using 3P Weibull distribution.

	Model 4			Model 5			Model 6		
L	OSB	*n*_*h*_	$\sum_{h=1}^{L}W_{h}\sigma_{h}$	OSB	*n*_*h*_	$\sum_{h=1}^{L}W_{h}\sigma_{h}$	OSB	*n*_*h*_	$\sum_{h=1}^{L}W_{h}\sigma_{h}$
2	9.56	28	0.0061	9.56	28	0.012	9.56	28	0.0140
		472			472			472	
3	7.37	4		7.37	4		7.37	4	
	11.84	194	0.0041	11.84	194	0.008	11.83	194	0.0096
		302			302			302	
4	6.30	2		6.30	2		6.30	2	
	9.56	38	0.0031	9.56	38	0.006	9.56	38	0.0073
	13.01	296		13.01	296		13.02	296	
		164			164			164	
5	5.66	2		5.66	2		5.66	2	
	8.24	10		8.23	10		8.24	10	
	10.92	99	0.0025	10.91	99	0.005	10.91	99	0.0058
	13.74	298		13.75	298		13.74	298	
		91			91			91	
6	5.24	2		5.24	2		5.24	2	
	7.37	4		7.37	4		7.37	4	
	9.56	35	0.0021	9.56	35	0.004	9.56	35	0.0049
	11.84	149		11.84	149		11.84	149	
	14.23	256		14.23	256		14.23	256	
		54			54			54	

Various other investigations related to OSB, sample size and the performance of the proposed technique, in both real and simulated data, are carried out and discussed in the following section and subsections.

## Results and discussion

Primarily, this paper involves the usage of multiple auxiliary variables in determining the OSB for the study variable. Investigations into the performance of the proposed method are also carried out to investigate some of the very pertinent issues such as:

1. Comparison of results using single and multiple auxiliary variables;
2. Comparison with other established methods of stratification in literature;
3. Determination of the optimum number of strata;
4. Comparison of stratification using other skewed distribution such as 3P Gamma;
5. Sensitivity of the proposed method with linear regression against nonlinear regression;
6. Consistency of the results obtained for real data with a simulated data set.

Thus, in the following subsections, comparative results are presented for the three models that are to create OSB for the main variable in real and simulated data. These are done to ascertain the effects of using a single auxiliary variable and multiple auxiliary variables in terms of the changes observed in the OSB, sample sizes and the ∑*W*_*h*_ *σ*_*h*_ values achieved for *L* = 2, …, 6. Together with results on the performance of the proposed method against other methods, results for 3P Gamma distribution and nonlinear regression are also presented.

### Use of single and multiple auxiliary variables

Tables 1 and 2 present the OSB, sample sizes and ∑*W*_*h*_ *σ*_*h*_ values for real and simulated data respectively. For real data in Model 2, which uses Folate, ∑*W*_*h*_ *σ*_*h*_ values are the lowest in the three models while for simulated data, it is lowest in Model 4 which uses variable *x*_1_. The ∑*W*_*h*_ *σ*_*h*_ values using the other models (i.e., models 1 & 3 in real data and 5 & 6 in simulated data) are close to each other. It is seen from the results that the ∑*W*_*h*_ *σ*_*h*_ values of the main variables in both real and simulated data appear to be declining exponentially as *L* increases in all the models. It must also be noted that in Table 2, the OSB and OSS are equivalent in all three models. This is due to the fact that the simulated data set is quite large and it results in a very precise-fitting of the distribution, which leads to equivalent OSB in all three models.

The findings in both data are similar in the sense that a single auxiliary variable model performs either better or worse than the model with multiple auxiliary variables. In real data, Model 2 performs better than Models 1 and 3 while in simulated data, Model 4 performs better than Models 5 and 6. This may be due to the fact that both Model 2 in real data and Model 4 in simulated data have a much weaker correlation with the dependent variable (see Tables 3 and 4). The results in Tables 3 and 4 presents key statistics such as the correlations, measure of regression error (RSE) and goodness of fit (AIC) for all the models in both data. It appears that the model with the auxiliary variable(s) that has the lowest correlation and Adjusted *R*^2^ and the highest RSE or AIC performs the best for the proposed method. Thus, the proposed method of stratification works best with uncorrelated auxiliary variable(s).

**Table 3:** Measure of Error, GoF and AIC for real data.

Model	Correlation	RSE	Adj *R*^2^	AIC
1	0.3498	1.566	12.11%	2707.66
2	0.1612	1.649	2.46%	2783.07
3	0.354	1.562	12.54%	2705.08

**Table 4:** Measure of Error, GoF and AIC for simulated data.

Model	Correlation	RSE	Adj *R*^2^	AIC
4	0.014	1.842	0.02%	20303.81
5	0.023	1.842	0.03%	20302.19
6	0.017	1.842	0.03%	20303.25

### Comparison with other available methods

For the purpose of the comparison of the performance of the proposed method, the following univariate methods available in the literature are considered:

1. Cum $\sqrt{f}$ method [20].
2. Geometric method of [19].
3. Lavallée and Hidiroglou (Kozak) method [11, 18]

The stratification package developed by [56] in the *R* statistical software is used to determine the OSB and sample sizes for the main study variable, Haemoglobin. The OSB are then used to compute the variance of the estimated mean (i.e., the values of the objective function or ∑*W*_*h*_ *σ*_*h*_) in each of the six models so that a comparative analysis could be carried out between the established methods and the proposed method. Note that comparisons are only possible here since the main variable is available to us in this example. The three methods above need the main variable to work out the OSB, however, the proposed method can work on auxiliary variable(s) to compute OSB for the main variables, with a few assumptions on the main variable.

The results, based on Models 1, 2 and 3 for real data, are given in Table 5, which presents the ∑*W*_*h*_ *σ*_*h*_ values of the estimate for Cum $\sqrt{f}$ method, Geometric method, Lavallée and Hidiroglou’s method and the proposed method with a fixed total sample of size *n* = 500, for *L* = 2, 3, …, 6. The efficiencies of the proposed DP method over the other three methods are also presented in the table.

**Table 5:** Comparison of $\sum_{h=1}^{L}W_{h}\sigma_{h}$ for different models in real data.

	**Model 1**				**Efficiency (%) of DP Over**		
L	**Cum $\sqrt{f}$**	**Geo**	**L-H (Kozak)**	**Prop. DP**	**Cum $\sqrt{f}$**	**Geo**	**L-H (Kozak)**
2	0.096	0.098	0.097	0.094	101.9	104.9	103.2
3	0.071	0.067	0.078	0.063	112.6	105.1	123.1
4	0.057	0.050	0.071	0.048	120.4	105.4	148.2
5	0.049	0.040	0.047	0.038	128.2	105.5	124.2
6	0.042	0.034	0.048	0.038	130.5	105.5	149.6
	**Model 2**				**Efficiency (%) of DP Over**		
2	0.024	0.028	0.024	0.024	101.3	116.3	101.3
3	0.018	0.019	0.020	0.017	102.6	111.5	113.7
4	0.014	0.015	0.018	0.013	109.3	112.2	135.1
5	0.012	0.012	0.013	0.010	116.57	112.5	121.1
6	0.010	0.010	0.013	0.009	119.86	112.7	144.8
	**Model 3**				**Efficiency (%) of DP Over**		
2	0.091	0.094	0.092	0.089	101.8	104.9	103.1
3	0.068	0.064	0.074	0.060	112.4	105.2	122.9
4	0.055	0.048	0.067	0.045	120.2	105.5	147.9
5	0.047	0.038	0.045	0.036	128.0	105.6	124.2
6	0.040	0.032	0.045	0.030	130.4	105.6	149.5

Upon examination of these results, it is noted that when a single auxiliary variable (Model 1) is used to determine OSB, the proposed method performs considerably well over the three methods and the efficiency of these OSB increases by about 2% to 50% for *L* = 1, 2, …, 6. Model 2 also produces much more efficient OSB over other methods and the efficiency increases from about 1% to 49%, which is quite similar to Model 1. Model 3 also increases the efficiencies from about 2% to 50%, being almost exactly similar to Model 1. Thus, with the use of auxiliary variables, either single or both, the proposed method increases the precision of the estimate compared to other univariate methods.

Table 6 provides the OSB and sample sizes using the other methods which can be compared with the results of the proposed method presented in Table 1.

**Table 6:** OSB and sample sizes for haemoglobin using other methods in real data.

	Cum $\sqrt{f}$		Geometric		L-H (Kozak)	
L	OSB	*n*_*h*_	OSB	*n*_*h*_	OSB	*n*_*h*_
2	12.15	255	10.15	39	12.35	284
		245		461		216
3	11.28	180	8.57	10	11.55	222
	13.23	181	12.03	192	12.75	64
		139		298		214
4	10.64	53	7.87	5	11.35	194
	12.15	46	10.15	39	12.35	42
	13.66	264	13.1	288	13.05	25
		137		168		239
5	10.2	81	7.48	1	9.25	37
	11.72	71	9.17	17	11.95	243
	12.8	56	11.24	91	12.75	43
	13.88	183	13.78	305	13.55	41
		109		86		136
6	9.77	36	7.23	1	9.35	41
	11.07	28	8.57	8	12.05	257
	12.15	35	10.15	33	12.65	27
	13.01	162	12.03	144	13.05	10
	14.09	146	14.26	260	13.55	15
		93		54		150

For simulated data, Table 7 presents the ∑*W*_*h*_ *σ*_*h*_ values for the three methods along with the proposed method for the three different models (Models 4-6) together with the efficiencies of the proposed method over the others. The results generally support the similar findings obtained for real data. Compared to all other methods, the proposed method increases the precision ranging from about 11% to 63% in Model 4 and 21% to 133% in Model 6. For Model 5, the proposed method increases the precision ranging from about 26% to 127% against Cum $\sqrt{f}$ method and 25% to 135% against L-H (Kozak) method. It doesn’t perform so well against Geometric method. Table 8 provides the OSB and sample sizes using the other methods which can be compared with the results of the proposed method presented in Table 2.

**Table 7:** Comparison of $\sum_{h=1}^{L}W_{h}\sigma_{h}$ for different models in simulated data.

	**Model 4**				**Efficiency (%) of DP Over**		
L	**Cum $\sqrt{f}$**	**Geo**	**L-H (Kozak)**	**Prop. DP**	**Cum $\sqrt{f}$**	**Geo**	**L-H (Kozak)**
2	0.0067	0.0071	0.0067	0.0061	110.88	116.53	109.91
3	0.0052	0.0049	0.0052	0.0041	127.66	119.93	127.53
4	0.0043	0.0038	0.0044	0.0031	137.62	121.83	140.42
5	0.0037	0.0031	0.0038	0.0025	145.74	122.58	151.81
6	0.0034	0.0026	0.0035	0.0021	156.65	122.17	163.05
	**Model 5**				**Efficiency (%) of DP Over**		
2	0.015	0.011	0.015	0.012	126.23	93.68	125.07
3	0.013	0.008	0.013	0.008	157.88	93.76	157.16
4	0.011	0.006	0.011	0.006	181.48	94.10	184.09
5	0.010	0.005	0.010	0.005	202.11	94.35	209.14
6	0.009	0.004	0.010	0.004	227.15	94.61	235.25
	**Model 6**				**Efficiency (%) of DP Over**		
2	0.017	0.014	0.017	0.014	120.90	98.00	119.89
3	0.014	0.010	0.014	0.010	148.97	99.39	148.48
4	0.012	0.007	0.013	0.007	169.85	100.06	172.45
5	0.011	0.006	0.011	0.006	188.30	100.47	182.40
6	0.010	0.005	0.011	0.005	210.60	100.75	233.03

**Table 8:** OSB and sample sizes for *y* using other methods in simulated data.

	Cum $\sqrt{f}$		Geometric		L-H (Kozak)	
L	OSB	*n*_*h*_	OSB	*n*_*h*_	OSB	*n*_*h*_
2	12.15	246	7.28	119	12.05	288
		254		381		212
3	11.06	123	5.51	8	11.02	149
	13.23	188	9.61	408	13.06	150
		189		84		201
4	10.24	90	4.79	2	10.33	144
	12.15	103	7.28	149	12.12	119
	13.78	152	11.05	310	13.62	107
		155		39		130
5	9.69	55	4.41	1	9.88	89
	11.33	123	6.16	34	11.45	89
	12.69	88	8.6	300	12.69	88
	14.05	99	12.01	144	13.93	96
		135		21		138
6	9.42	65	4.17	1	9.61	75
	11.06	60	5.51	9	11.08	61
	12.15	68	7.28	153	12.21	71
	13.23	122	9.61	249	13.19	67
	14.32	97	12.7	75	14.24	83
		88		13		143

When considering Weibull distribution cases, the sample allocations under the proposed method (which uses Neyman allocation given by (19)) are given in Tables 1 and 2 for real and simulated data respectively. In the method, the overall size of strata (*N*_*h*_) as well as variability ($S_{h}^{2}$) of the auxiliary variable(s) affects the stratum sample sizes (*n*_*h*_), i.e., *n*_*h*_ ∝ *W*_*h*_ *S*_*h*_. It is noticeable that for both real and simulated examples, the stratum samples sizes given by the proposed method is a bit different from the sample sizes given by other methods presented in Tables 6 and 8. This is because of the differences seen in the OSB, and hence the *W*_*h*_, between the methods.

To substantiate the results, the method of bootstrap re-sampling is used to investigate the behaviour of the findings made earlier on the real dataset. A large number (*n* = 10,000) of independent re-samples are drawn with replacement from the population data. The re-samples are of the same size as the Anaemia data (*N* = 724), creating many variants of the original data. Since there are three variables in the Anaemia population, bootstrap re-sampling is done on individuals, which means three variables are randomly generated for each population. From the large number of bootstrap re-samples, results for only 5 randomly selected samples are presented for the sake of brevity. We consider all three models given by equations in (39).

For all five bootstrap samples, Tables 9—13 present the OSB, OSS (*n*_*h*_) and variances $\left(\sum_{h=1}^{L}W_{h}\sigma_{h}\right)$ for all three models are calculated using the proposed method. It is again observed that Model 2 has the lowest variance and this means that it is the best model to use out of the three. To further investigate why Model 2 is the best, Table 14 is drawn up. It is found out that results are consistent in all five bootstrap samples. Model 2 performs the best because it has a low correlation with the main variable together with a high RSE, a very low adjusted *R*^2^ and the highest AIC amongst the three models. Thus, whether it is a single or multiple auxiliary variables (ie., all models studied herein) used in the formulation of the problem of stratification, the gains in efficiency of the proposed method over other established methods are substantial. These are given by Tables 15—19 where we see that the variances given by the proposed method are lower than the other methods. Hence, with bootstrap re-sampling procedure, it is seen that we obtain consistent findings to what was seen in the original Anaemia data.

**Table 9:** Results for bootstrap re-sample 1 using 3P Weibull distribution.

	Model 1			Model 2			Model 3		
L	OSB	*n*_*h*_	$\sum_{h=1}^{L}W_{h}\sigma_{h}$	OSB	*n*_*h*_	$\sum_{h=1}^{L}W_{h}\sigma_{h}$	OSB	*n*_*h*_	$\sum_{h=1}^{L}W_{h}\sigma_{h}$
2	11.05	100	0.086	11.18	113	0.019	11.05	100	0.084
		400			387			400	
3	9.18	19	0.058	9.37	28	0.013	9.19	19	0.057
	12.85	311		12.97	311		12.86	311	
		170			161			170	
4	8.36	12	0.044	8.52	16	0.010	8.37	12	0.043
	10.95	74		11.13	89		10.96	74	
	13.81	355		13.91	341		13.82	355	
		59			54			59	
5	7.89	6	0.035	8.03	12	0.008	7.89	6	0.034
	9.87	33		10.06	34		9.88	33	
	12.06	157		12.22	174		12.07	157	
	14.40	269		14.49	244		14.41	269	
		35			37			35	
6	7.58	4	0.029	7.70	5	0.007	7.59	4	0.029
	9.18	13		9.37	21		9.19	13	
	10.94	73		11.12	85		10.95	73	
	12.82	206		12.96	194		12.83	206	
	14.81	190		14.88	180		14.81	190	
		15			15			15	

**Table 10:** Results for bootstrap re-sample 2 using 3P Weibull distribution.

	Model 1			Model 2			Model 3		
L	OSB	*n*_*h*_	$\sum_{h=1}^{L}W_{h}\sigma_{h}$	OSB	*n*_*h*_	$\sum_{h=1}^{L}W_{h}\sigma_{h}$	OSB	*n*_*h*_	$\sum_{h=1}^{L}W_{h}\sigma_{h}$
2	10.95	101	0.106	11.09	115	0.034	11.17	121	0.099
		399			385			379	
3	9.10	27	0.072	9.20	27	0.023	9.30	31	0.067
	12.79	273		12.88	284		12.92	294	
		200			189			175	
4	8.30	11	0.054	8.39	11	0.018	8.50	16	0.050
	10.86	74		10.98	87		11.05	95	
	13.76	333		13.83	330		13.86	314	
		82			72			75	
5	7.84	12	0.043	7.92	13	0.014	8.04	14	0.040
	9.78	25		9.89	26		9.98	25	
	11.98	143		12.09	158		12.14	180	
	14.36	268		14.42	253		14.45	230	
		52			50			51	
6	7.54	8	0.036	7.61	11	0.012	7.73	12	0.034
	9.11	16		9.21	14		9.30	16	
	10.85	54		10.96	65		11.03	74	
	12.75	203		12.84	195		12.89	175	
	14.77	189		14.82	189		14.84	196	
		30			26			27	

**Table 11:** Results for bootstrap re-sample 3 using 3P Weibull distribution.

	Model 1			Model 2			Model 3		
L	OSB	*n*_*h*_	$\sum_{h=1}^{L}W_{h}\sigma_{h}$	OSB	*n*_*h*_	$\sum_{h=1}^{L}W_{h}\sigma_{h}$	OSB	*n*_*h*_	$\sum_{h=1}^{L}W_{h}\sigma_{h}$
2	10.85	83	0.110	11.00	96	0.022	10.85	83	0.106
		417			404			417	
3	8.96	20	0.074	9.21	25	0.015	8.96	20	0.072
	12.59	258		12.74	277		12.59	258	
		222			198			222	
4	8.19	8	0.056	8.40	12	0.011	8.19	8	0.054
	10.69	55		10.93	91		10.70	55	
	13.54	339		13.66	306		13.54	339	
		98			91			98	
5	7.75	7	0.045	7.93	9	0.009	7.75	7	0.043
	9.63	28		9.88	31		9.63	28	
	11.79	119		12.00	141		11.79	119	
	14.12	295		14.23	271		14.12	295	
		51			48			51	
6	7.46	2	0.037	7.62	8	0.008	7.46	2	0.036
	8.97	15		9.21	14		8.97	15	
	10.68	48		10.92	81		10.68	48	
	12.55	197		12.72	173		12.55	197	
	14.52	198		14.61	192		14.52	198	
		40			32			40	

**Table 12:** Results for bootstrap re-sample 4 using 3P Weibull distribution.

	Model 1			Model 2			Model 3		
L	OSB	*n*_*h*_	$\sum_{h=1}^{L}W_{h}\sigma_{h}$	OSB	*n*_*h*_	$\sum_{h=1}^{L}W_{h}\sigma_{h}$	OSB	*n*_*h*_	$\sum_{h=1}^{L}W_{h}\sigma_{h}$
2	11.17	105	0.092	11.28	111	0.022	11.17	105	0.089
		395			389			395	
3	9.31	18	0.062	9.48	21	0.015	9.30	18	0.060
	12.93	315		13.03	325		12.92	315	
		167			153			167	
4	8.50	9	0.047	8.65	10	0.011	8.50	7	0.045
	11.05	90		11.22	108		11.05	98	
	13.87	334		13.96	323		13.86	329	
		67			59			66	
5	8.04	6	0.037	8.17	7	0.009	8.04	6	0.036
	9.99	33		10.16	35		9.98	33	
	12.14	183		12.29	180		12.14	183	
	14.45	233		14.53	242		14.45	233	
		45			36			45	
6	7.74	3	0.031	7.85	5	0.008	7.73	3	0.030
	9.31	18		9.48	18		9.30	18	
	11.04	79		11.21	92		11.03	79	
	12.89	190		13.02	199		12.89	190	
	14.84	189		14.91	170		14.84	189	
		21			17			21	

**Table 13:** Results for bootstrap re-sample 5 using 3P Weibull distribution.

	Model 1			Model 2			Model 3		
L	OSB	*n*_*h*_	$\sum_{h=1}^{L}W_{h}\sigma_{h}$	OSB	*n*_*h*_	$\sum_{h=1}^{L}W_{h}\sigma_{h}$	OSB	*n*_*h*_	$\sum_{h=1}^{L}W_{h}\sigma_{h}$
2	11.17	93	0.092	11.28	98	0.022	11.17	93	0.089
		407			402			407	
3	9.31	20	0.062	9.48	20	0.015	9.30	18	0.060
	12.93	295		13.03	309		12.92	299	
		185			171			183	
4	8.50	8	0.047	8.65	10	0.011	8.50	8	0.045
	11.05	85		11.22	90		11.05	85	
	13.87	334		13.96	325		13.86	334	
		73			75			73	
5	8.04	6	0.037	8.17	6	0.009	8.04	6	0.036
	9.99	31		10.16	37		9.98	31	
	12.14	141		12.29	154		12.14	141	
	14.45	282		14.53	261		14.45	282	
		40			42			40	
6	7.74	4	0.031	7.85	4	0.008	7.73	3	0.030
	9.31	17		9.48	18		9.30	19	
	11.04	63		11.21	73		11.03	63	
	12.89	203		13.02	189		12.89	203	
	14.84	193		14.91	196		14.84	192	
		20			20			20	

**Table 14:** Measure of Error, GoF and AIC for bootstrap samples of Anaemia data.

	**Bootstrap Sample 1**			
Model	Correlation	RSE	Adj *R*^2^	AIC
1	0.3340	1.5330	0.11	2677.05
2	0.1239	1.6140	0.01	2751.50
3	0.3320	1.5330	0.11	2678.18
	**Bootstrap Sample 2**			
Model	Correlation	RSE	Adj *R*^2^	AIC
1	0.400	1.560	0.16	2702.64
2	0.211	1.664	0.04	2795.95
3	0.409	1.552	0.17	2695.83
	**Bootstrap Sample 3**			
Model	Correlation	RSE	Adj *R*^2^	AIC
1	0.394	1.555	0.15	2697.92
2	0.157	1.671	0.02	2801.71
3	0.398	1.551	0.16	2695.05
	**Bootstrap Sample 4**			
Model	Correlation	RSE	Adj *R*^2^	AIC
1	0.356	1.534	0.13	2678.26
2	0.144	1.624	0.02	2761.15
3	0.361	1.529	0.13	2674.93
	**Bootstrap Sample 5**			
Model	Correlation	RSE	Adj *R*^2^	AIC
1	0.334	1.535	0.11	2678.95
2	0.194	1.598	0.04	2737.13
3	0.346	1.527	0.12	2672.31

**Table 15:** Comparison of $\sum_{h=1}^{L}W_{h}\sigma_{h}$ for different models in bootstrap sample 1.

	**Model 1**				**Efficiency (%) of DP Over**		
L	**Prop. DP**	**Cum $\sqrt{f}$**	**Geo**	**L-H (Kozak)**	**Cum $\sqrt{f}$**	**Geo**	**L-H (Kozak)**
2	0.086	0.089	0.091	0.088	102.69	105.32	101.44
3	0.058	0.065	0.062	0.065	111.77	105.63	111.35
4	0.044	0.053	0.046	0.052	122.06	105.90	118.85
5	0.035	0.045	0.037	0.046	127.14	106.01	131.65
6	0.029	0.040	0.031	0.042	135.84	106.07	141.68
	**Model 2**				**Efficiency (%) of DP Over**		
2	0.019	0.018	0.021	0.018	96.29	110.30	96.24
3	0.013	0.013	0.014	0.014	103.37	111.98	105.80
4	0.010	0.011	0.011	0.011	113.94	112.69	110.74
5	0.008	0.009	0.009	0.010	117.47	113.03	123.29
6	0.007	0.008	0.007	0.009	124.87	113.18	132.10
	**Model 3**				**Efficiency (%) of DP Over**		
2	0.084	0.086	0.089	0.085	102.68	105.36	101.43
3	0.057	0.063	0.060	0.063	111.77	105.68	111.36
4	0.043	0.052	0.045	0.051	122.06	105.96	118.85
5	0.034	0.043	0.036	0.045	127.14	106.07	131.66
6	0.029	0.039	0.030	0.040	135.84	106.13	141.69

**Table 16:** Comparison of $\sum_{h=1}^{L}W_{h}\sigma_{h}$ for different models in bootstrap sample 2.

	**Model 1**				**Efficiency (%) of DP Over**		
L	**Prop. DP**	**Cum $\sqrt{f}$**	**Geo**	**L-H (Kozak)**	**Cum $\sqrt{f}$**	**Geo**	**L-H (Kozak)**
2	0.106	0.108	0.112	0.108	101.13	105.01	101.69
3	0.072	0.081	0.076	0.081	113.26	105.22	112.61
4	0.054	0.066	0.057	0.062	121.58	105.49	114.77
5	0.043	0.057	0.046	0.054	131.91	105.59	124.67
6	0.036	0.049	0.038	0.050	135.30	105.65	138.48
	**Model 2**				**Efficiency (%) of DP Over**		
2	0.034	0.033	0.038	0.033	95.62	109.59	95.60
3	0.023	0.024	0.026	0.025	104.81	110.49	105.55
4	0.018	0.020	0.020	0.019	112.39	111.16	106.94
5	0.014	0.017	0.016	0.016	119.23	111.42	116.14
6	0.012	0.015	0.013	0.015	123.05	111.54	128.61
	**Model 3**				**Efficiency (%) of DP Over**		
2	0.099	0.101	0.105	0.102	101.56	105.72	102.11
3	0.067	0.076	0.071	0.076	113.97	106.26	113.37
4	0.050	0.062	0.054	0.058	122.47	106.64	115.63
5	0.040	0.054	0.043	0.051	132.85	106.79	125.67
6	0.034	0.046	0.036	0.047	136.31	106.86	139.59

**Table 17:** Comparison of $\sum_{h=1}^{L}W_{h}\sigma_{h}$ for different models in bootstrap sample 3.

	**Model 1**				**Efficiency (%) of DP Over**		
L	**Prop. DP**	**Cum $\sqrt{f}$**	**Geo**	**L-H (Kozak)**	**Cum $\sqrt{f}$**	**Geo**	**L-H (Kozak)**
2	0.110	0.112	0.115	0.111	102.55	104.94	101.66
3	0.074	0.083	0.078	0.085	111.02	104.63	113.91
4	0.056	0.065	0.059	0.065	116.68	104.97	116.48
5	0.045	0.055	0.047	0.058	123.54	105.02	130.36
6	0.037	0.047	0.039	0.054	126.22	105.02	143.78
	**Model 2**				**Efficiency (%) of DP Over**		
2	0.022	0.021	0.024	0.021	95.43	109.97	95.44
3	0.015	0.015	0.017	0.015	102.21	111.39	103.28
4	0.011	0.012	0.013	0.012	105.62	112.22	106.53
5	0.009	0.010	0.010	0.011	111.96	112.45	117.76
6	0.008	0.009	0.009	0.009	115.04	112.57	124.99
	**Model 3**				**Efficiency (%) of DP Over**		
2	0.106	0.108	0.111	0.107	102.46	104.96	101.57
3	0.072	0.080	0.075	0.082	110.93	104.69	113.79
4	0.054	0.063	0.057	0.063	116.57	105.04	116.38
5	0.043	0.053	0.045	0.056	123.43	105.09	130.23
6	0.036	0.046	0.038	0.052	126.12	105.10	143.59

**Table 18:** Comparison of $\sum_{h=1}^{L}W_{h}\sigma_{h}$ for different models in bootstrap sample 4.

	**Model 1**				**Efficiency (%) of DP Over**		
L	**Prop. DP**	**Cum $\sqrt{f}$**	**Geo**	**L-H (Kozak)**	**Cum $\sqrt{f}$**	**Geo**	**L-H (Kozak)**
2	0.092	0.094	0.096	0.094	102.07	104.53	102.13
3	0.062	0.070	0.065	0.071	112.87	104.74	113.48
4	0.047	0.056	0.049	0.056	120.54	104.90	119.18
5	0.037	0.046	0.039	0.046	123.22	105.01	123.40
6	0.031	0.040	0.033	0.043	127.54	105.04	138.27
	**Model 2**				**Efficiency (%) of DP Over**		
2	0.022	0.021	0.024	0.021	96.45	109.56	96.45
3	0.015	0.016	0.017	0.016	103.61	110.97	106.61
4	0.011	0.013	0.013	0.013	110.29	111.50	110.04
5	0.009	0.010	0.010	0.010	114.59	105.58	114.37
6	0.008	0.009	0.010	0.010	115.82	134.02	128.56
	**Model 3**				**Efficiency (%) of DP Over**		
2	0.089	0.091	0.093	0.091	101.94	104.58	102.00
3	0.060	0.068	0.063	0.068	112.63	104.79	113.28
4	0.045	0.055	0.048	0.054	120.29	104.97	118.95
5	0.036	0.045	0.038	0.045	123.00	105.08	123.18
6	0.030	0.039	0.032	0.042	127.26	105.12	138.04

**Table 19:** Comparison of $\sum_{h=1}^{L}W_{h}\sigma_{h}$ for different models in bootstrap sample 5.

	**Model 1**				**Efficiency (%) of DP Over**		
L	**Prop. DP**	**Cum $\sqrt{f}$**	**Geo**	**L-H (Kozak)**	**Cum $\sqrt{f}$**	**Geo**	**L-H (Kozak)**
2	0.086	0.088	0.090	0.087	103.00	105.03	101.69
3	0.058	0.065	0.061	0.065	112.47	105.48	111.91
4	0.043	0.053	0.046	0.052	122.85	105.75	119.63
5	0.035	0.045	0.037	0.046	128.09	105.87	132.56
6	0.029	0.040	0.031	0.041	136.94	105.93	142.75
	**Model 2**				**Efficiency (%) of DP Over**		
2	0.028	0.028	0.032	0.028	99.40	113.65	99.33
3	0.020	0.020	0.022	0.021	102.73	110.98	105.09
4	0.015	0.017	0.016	0.016	113.20	111.65	110.02
5	0.012	0.014	0.013	0.015	116.70	111.95	122.46
6	0.010	0.012	0.011	0.013	124.05	112.08	131.20
	**Model 3**				**Efficiency (%) of DP Over**		
2	0.080	0.082	0.084	0.081	102.70	105.14	101.45
3	0.054	0.060	0.057	0.060	112.03	105.62	111.59
4	0.040	0.050	0.043	0.048	122.43	105.93	119.22
5	0.032	0.041	0.034	0.043	127.62	106.06	132.14
6	0.027	0.037	0.029	0.038	136.42	106.12	142.28

### Number of strata

**Fig 4:**
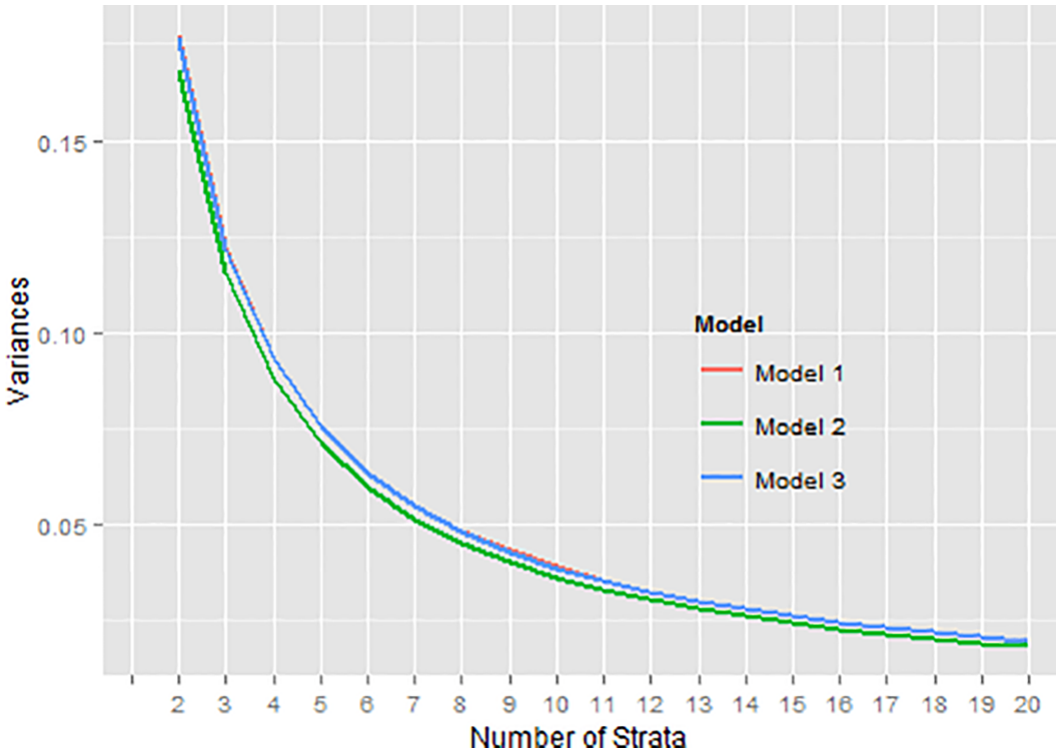
$Var({\bar{y}}_{st})$ for haemoglobin in real data.

**Fig 5:**
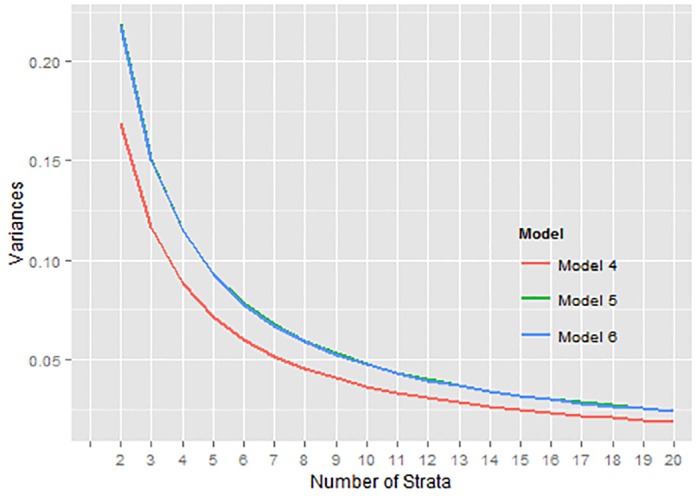
$Var({\bar{y}}_{st})$ for *y* in simulated data.

To study the relationship between the number of strata and the ∑*W*_*h*_ *σ*_*h*_ value, an investigation is carried out for the real and simulated data using the six models. The ∑*W*_*h*_ *σ*_*h*_ are calculated using the proposed method and the results are presented for *L* = 2, 3, …, 20. These are presented in Figs 4 and 5 where the curves appear to be on top of each other and all of them decrease exponentially. After *L* = 7, where the “*elbow*” is found, the rate of decrease in the ∑*W*_*h*_ *σ*_*h*_ values from there onwards is not as big as what is seen from *L* = 2 to 7. For argument’s sake, one might even be comfortable with *L* = 6 as the appropriate number of strata. The finding supports the investigation carried out by Cochran [1] that the number of strata to be constructed beyond six is not much useful in terms of the relative gain in efficiency or the reduction of ∑*W*_*h*_ *σ*_*h*_. All six models in real and simulated data are very similar when it comes to the relative gain in efficiencies and one can easily pick out *L* = 7 where the “*elbow*” appears, indicating that the percentage reduction thereafter is not worth investing in for a sample survey since additional costs are involved with increase in the number of strata. Increasing the number of strata to more than 7 may not be a good trade-off for a little gain of precision in the estimates.

### Using skewed distributions other than Weibull

The distribution of the auxiliary variable can vary depending on how well the data fits a particular skewed distribution based on the best MLE of its parameters. Weibull is selected in this paper due it’s versatility in fitting skewed distributions, especially for health data. To probe into the performance of Weibull distribution against any other skewed distribution, both auxiliary variables in the real and simulated data are fitted with a 3P Gamma distributions because of its moderately skewed profile as well. Three different linear regression models are again used for the comparison of results. The associated MPP is formulated and solved using the DP technique.

The OSB, sample sizes and ∑*W*_*h*_ *σ*_*h*_ values are presented in Table 20 for the real data while Table 21 is for the simulated data. Similar to the results obtained under Weibull distribution, the results for Gamma show that the OSB are slightly different from each other in all the three models. To compare the performance of Gamma results against those obtained under Weibull distribution for the real data, ∑*W*_*h*_ *σ*_*h*_ values from Table 20 are compared with Table 1. They reveal that fitting the data with Weibull distribution yields a much more efficient set of OSB compared to fitting the data with Gamma distribution. This holds true for both single or multiple auxiliary variables. Results for the simulated data in Tables 2 and 21 also reveal similar findings. This may be due to the fact that Weibull was a better fit than Gamma for the two auxiliary variables,

**Table 20:** Results for real data using 3P gamma distribution.

	Model 1			Model 2			Model 3		
L	OSB	*n*_*h*_	$\sum_{h=1}^{L}W_{h}\sigma_{h}$	OSB	*n*_*h*_	$\sum_{h=1}^{L}W_{h}\sigma_{h}$	OSB	*n*_*h*_	$\sum_{h=1}^{L}W_{h}\sigma_{h}$
2	11.08	107	0.095	11.17	116	0.0281	11.08	107	0.091
		393			384			393	
3	9.23	22		9.32	25		9.22	22	
	12.87	306	0.064	12.94	314	0.0194	12.87	306	0.061
		172			161			172	
4	8.38	9		8.46	11		8.38	9	
	10.99	93	0.0482	11.08	102	0.0147	10.98	93	0.046
	13.83	336		13.88	321		13.83	335	
		64			66			64	
5	7.90	7		7.97	7		7.89	6	
	9.90	40		10.00	44		9.90	40	
	12.09	157	0.0386	12.17	169	0.0118	12.09	157	0.037
	14.42	257		14.46	240		14.42	257	
		39			40			39	
6	7.58	5		7.64	5		7.58	5	
	9.21	20		9.3	22		9.2	20	
	10.97	75	0.0322	11.07	88	0.0099	10.97	80	0.031
	12.85	205		12.92	203		12.84	203	
	14.82	174		14.86	161		14.82	173	
		20			21			20	

**Table 21:** Results for simulated data using 3P gamma distribution.

	Model 4			Model 5			Model 6		
L	OSB	*n*_*h*_	$\sum_{h=1}^{L}W_{h}\sigma_{h}$	OSB	*n*_*h*_	$\sum_{h=1}^{L}W_{h}\sigma_{h}$	OSB	*n*_*h*_	$\sum_{h=1}^{L}W_{h}\sigma_{h}$
2	2.48	28	0.4117	4.28	21	0.2393	2.49	21	0.4077
		472			479			479	
3	2.53	4		2.55	2		2.53	2	
	6.01	194	0.4069	5.45	203	0.2200	6.00	203	0.4052
		302			295			295	
4	2.16	2		2.07	2		2.17	2	
	2.99	38	0.3290	4.07	26	0.1857	2.99	26	0.3289
	5.95	296		6.25	325		5.94	325	
		164			147			147	
5	1.98	2		1.77	2		1.98	2	
	2.60	10		3.17	10		2.61	10	
	3.37	99	0.2822	4.88	99	0.1601	3.38	99	0.2829
	5.98	298		6.69	298		5.98	298	
		91			91			91	
6	1.96	2		1.56	2		1.96	2	
	2.56	4		2.64	4		2.56	4	
	3.28	35	0.2766	3.99	35	0.1387	3.28	35	0.2767
	5.07	149		5.45	149		5.07	149	
	6.79	256		6.99	256		6.79	256	
		54			54			54	

### Linear versus nonlinear regression

As shown in (5), the proposed method can incorporate linear as well as nonlinear regression for construction of OSB. In the preceding sections, it has been discussed that linear regression performs well in real as well as simulated data. To investigate the sensitiveness of linear regression over nonlinear regression, a simple case of quadratic regression is fitted in this section. Consider that the study variables are to be stratified using a single auxiliary variable (e.g., Iron in real & and *x*_2_ in simulated data). Then, *λ*(*x*) in (5) reduces to:

$$\begin{array}{ccc} & Real: & \lambda \left(x\right)=\beta_{0}+\beta_{1}Iron+\beta_{2}Iron^{2} \end{array}$$ (40)

$$\begin{array}{ccc} & Simulated: & \lambda \left(x\right)=\beta_{0}+\beta_{1}x_{2}+\beta_{2}x_{2}^{2} \end{array}$$ (41)

The ANOVA results for this quadratic regression reveals that the model is statistically significant (p-value < 0.001) for both real and simulated data.

Using the procedures discussed in Sections 3–7, the OSB and sample sizes are determined. Table 22 presents the results along with the ∑*W*_*h*_ *σ*_*h*_ values for real and simulated data respectively. The results reveal that for both data, the ∑*W*_*h*_ *σ*_*h*_ values from linear regression (Model 3 from Table 1 and Model 6 from Table 2) are lower than non-linear regression model which means that linear regression performs better than the nonlinear regression.

**Table 22:** Non-linear regression results for real and simulated data.

	Real Data			Simulated Data		
L	OSB	*n*_*h*_	$\sum_{h=1}^{L}W_{h}\sigma_{h}$	OSB	*n*_*h*_	$\sum_{h=1}^{L}W_{h}\sigma_{h}$
2	10.76	72	0.095	4.08	84	0.242
		428			416	
3	8.93	18		2.3	23	
	12.68	282	0.064	5.44	257	0.208
		200			220	
4	8.17	7		1.89	12	
	10.68	60	0.048	3.50	72	0.175
	13.66	351		5.96	308	
		82			108	
5	7.73	5		1.64	7	
	9.61	29		2.81	30	
	11.83	147	0.039	4.61	133	0.150
	14.28	270		6.54	272	
		49			58	
6	7.45	2		1.44	7	
	8.95	18		2.37	14	
	10.67	44	0.032	3.53	59	0.128
	12.62	213		5.13	170	
	14.7	194		6.82	213	
		29			37	

To investigate this further, Table 23 presents some key statistical measures such as measure of regression error (RSE) and goodness of fit (AIC) with regards to how the model under nonlinear regression performs against the models under linear regression for both real and simulated data. The measures for linear regression are presented in Tables 3 and 4. They reveal that the results are consistent with the findings earlier in the paper—that the model with the lowest Adjusted *R*^2^ and the highest RSE or AIC performs the best. Thus, linear regression model performs better than the nonlinear regression model.

**Table 23:** Measure of RSE and AIC for nonlinear regression models.

Data	RSE	Adj *R*^2^	AIC
Real	1.524	16.67%	2670.09
Simulated	0.734	5.46%	11104.42

## Conclusion

Stratified random sampling is an efficient and widely used sampling technique in health surveys to estimate the prevalence of diseases and many other parameters. Often, the surveyors encounter two major difficulties prior to drawing the samples and these are: (i) constructing the optimum strata within which the units are as homogeneous as possible and (ii) determining the optimum sample size to be drawn from each stratum, so that the precision of the estimates of parameters of the study or target variables are maximized. In this paper, a parametric-based method is proposed to address these two problems, which can be used to estimate parameters with more precision.

The optimum stratification based on the study variable is not feasible in practice since it is unknown prior to conducting the survey. Thus, the proposed technique uses auxiliary information in designing the sampling plan. This paper investigates how the usage of one or more auxiliary variables influence the OSB and hence the effect on the efficiency of the stratum boundaries by fitting a distribution of Weibull family that characterize many health variables. It also investigates the sensitivity of the OSB and the performance of the proposed method by fitting with other skewed distributions such as Gamma. Together with investigating the optimum number of strata, the proposed method also sees the sensitiveness of linear and nonlinear regression modelling techniques in implementing the proposed method.

The problem of finding the OSB is formulated as an MPP that seeks minimization of the variance of the estimated population parameter and solved using a DP technique. The solution procedure is implemented through a C++ computer program and an R script to facilitate the computation of the OSB through the C++ program. Both materials can be made available on request from the authors. After obtaining the OSB, they are then used to compute the optimum sample size for each stratum using Neyman allocation. Numerical examples using a real data set and a simulated data set are presented to illustrate the application, the sensitivity and the usefulness of the proposed technique. This paper also presents the results from cum $\sqrt{f}$ method [20], geometric method [19] and the generalized Lavallée and Hidiroglou’s method [11, 18] for a comparative analysis.

It can be concluded that in the construction of strata for health populations, usage of both single or multiple auxiliary variables leads to substantial gains in the precision of the estimates over other available methods. It was also established that using uncorrelated auxiliary variable(s) to determine OSB for the main variable leads to much more efficient results. It was also found out that when another skewed distribution such as Gamma is used to characterize the distribution of the auxiliary variables, it performed well but not quite as accurate as Weibull. Hence, the best-fit distribution should always be chosen for more accurate calculation of OSB. It was also found out that when linear regression was used in formulating the problem of stratification, it performed better than nonlinear regression. This simply depends on the data and one must always choose the best regression technique to represent the relationship between the variables.

## Appendix A

The following steps are followed in implementing the DP technique to solve the MPP for the OSB:

1. Start at *k* = 1. Set Φ_0_(*d*_0_) = 0.
2. Calculate Φ_1_(*d*_1_), the minimum value of RHS of (18) for *l*_1_ = *d*_1_, 0 ≤ *l*_1_ ≤ *d*_1_, and 0 ≤ *d*_1_ ≤ *d*.
3. Record Φ_1_(*d*_1_) and *l*_1_.
4. For *k* = 2, express the state variable as *d*_*k*−1_ = *d*_*k*_ − *l*_*k*_.
5. Set Φ_*k*_(*d*_*k*_) = 0 if *l*_*k*_ > *d*_*k*_, where 0 ≤ *d*_*k*_ ≤ *d*.
6. Calculate Φ_*k*_(*d*_*k*_), the minimum value of RHS of (17) for *l*_*k*_;0 ≤ *l*_*k*_ ≤ *d*_*k*_.
7. Record Φ_*k*_(*d*_*k*_) and *l*_*k*_.
8. For *k* ≥ 3, …, *L*, go to Step 4.
9. At *k* = *L*, Φ_*L*_(*d*) is obtained and hence the optimum value $l_{L}^{*}$ of *l*_*L*_ is obtained.
10. At *k* = *L* − 1, using the backward calculation for $d_{L-1}=d-l_{L}^{*}$, read the value of Φ_*L*−1_(*d*_*L*−1_) and hence the optimum value $l_{L-1}^{*}$ of *l*_*L*−1_.
11. Repeat Step 10 until the optimum value $l_{1}^{*}$ of *l*_1_ is obtained from Φ_1_(*d*_1_).

## References

[1] Cochran WG. (1977); Sampling techniques. New York, Wiley and Sons 1977;98:259–261.

[2] Lohr S. Sampling: design and analysis. Nelson Education; 2009.

[3] Dalenius T. The problem of optimum stratification. Scandinavian Actuarial Journal. 1950;(3-4):203–213. doi: 10.1080/03461238.1950.10432042

[4] Dalenius T, Gurney M. The problem of optimum stratification. II. Scandinavian Actuarial Journal. 1951;1951(1-2):133–148. doi: 10.1080/03461238.1951.10432134

[5] Mahalanobis PC. Some aspects of the design of sample surveys. SankhyÄ: The Indian Journal of Statistics. 1952; p. 1–7.

[6] Hansen MH, Hurwitz WN. On the Theory of Sampling from Finite Populations. The Annals of Mathematical Statistics. 1943;14(4):333–362. doi: 10.1214/aoms/1177731356

[7] Aoyama H. A study of the stratified random sampling. Annals of the Institute of Statistical Mathematics. 1954;6(1):1–36. doi: 10.1007/BF02960514

[8] Ekman G. An Approximation Useful in Univariate Stratification. The Annals of Mathematical Statistics. 1959;30(1):219–229. doi: 10.1214/aoms/1177706377

[9] Sethi VK. A note on optimum stratification of populations for estimating the population means. Australian Journal of Statistics. 1963;5(1):20–33. doi: 10.1111/j.1467-842X.1963.tb00134.x

[10] Unnithan VKG. The minimum variance boundry points of stratification. Sankhya. 1978;40(C):60–72.

[11] Lavallée P, Hidiroglou M. On the stratification of skewed populations. Survey methodology. 1988;14(1):33–43.

[12] Hidiroglou MA, Srinath KP. Problems associated with designing subannual business surveys. Journal of Business & Economic Statistics. 1993;11(4):397–405. doi: 10.1080/07350015.1993.10509973

[13] Sweet EM, Sigman RS. Evaluation of model-assisted procedures for stratifying skewed populations using auxiliary data. In: Proceedings of the Section on Survey Research Methods. vol. 1; 1995. p. 491–496.

[14] Rivest LP. A generalization of the Lavallée and Hidiroglou algorithm for stratification in business surveys. Survey Methodology. 2002;28(2):191–198.

[15] Niemiro W. Optimal construction of strata using random search method. Wiadomosci statystyczne. 1999;10:1–9.

[16] Nelder JA, Mead R. A simplex method for function minimization. The computer journal. 1965;7(4):308–313. doi: 10.1093/comjnl/7.4.308

[17] Lednicki B, Wieczorkowski R. Optimal stratification and sample allocation between subpopulations and strata. Statistics in transition. 2003;6(2):287–305.

[18] Kozak M. Optimal stratification using random search method in agricultural surveys. Statistics in Transition. 2004;6(5):797–806.

[19] Gunning P, Horgan JM. A new algorithm for the construction of stratum boundaries in skewed populations. Survey Methodology. 2004;30(2):159–166.

[20] Dalenius T, Hodges JL Jr. Minimum variance stratification. Journal of the American Statistical Association. 1959;54(285):88–101. doi: 10.1080/01621459.1959.10501501

[21] Horgan JM. Stratification of Skewed Populations: A review. International Statistical Review. 2006;74(1):67–76. doi: 10.1111/j.1751-5823.2006.tb00161.x

[22] Kozak M, Verma MR. Geometric versus optimization approach to stratification: A comparison of efficiency. Survey Methodology. 2006;32(2):157.

[23] Kozak M, Verma MR, Zielinski A. Modern approach to optimum stratification: Review and perspectives. Statistics in Transition. 2007;8(2):223–250.

[24] Khan EA, Khan MGM, Ahsan MJ. Optimum stratification: a mathematical programming approach. Calcutta Statistical Association Bulletin. 2002;52:323–333. doi: 10.1177/0008068320020518

[25] Khan MGM, Sehar N, Ahsan MJ. Optimum stratification for exponential study variable under Neyman allocation. Journal of the Indian Society of Agricultural Statistics. 2005;59(2):146–150.

[26] Khan MGM, Ahmad N, Khan S. Determining the Optimum Stratum Boundaries Using Mathematical Programming. Journal of Mathematical Modelling and Algorithms. 2009;8(4):1–15. doi: 10.1007/s10852-009-9115-3

[27] Nand N. Determining the Optimum Strata Boundary Points using Mathematical Programming. Survey Methodology. 2003;34(2):1–3.

[28] Khan MGM, Nand N, Ahmad N. Determining the optimum strata boundary points using dynamic programming. Survey Methodology. 2008;34(2):205–214.

[29] Nand N, Khan MGM. Optimum Stratification for Cauchy and Power Type Study Variable. Journal of Applied Statistical Science. 2009;16(4):453.

[30] Khan MGM, Reddy KG, Rao DK. Designing stratified sampling in economic and business surveys. Journal of Applied Statistics. 2015; p. 1–20.25484482

[31] Bühler W, Deutler T. Optimal stratification and grouping by dynamic programming. Metrika. 1975;22(1):161–175. doi: 10.1007/BF01899725

[32] Lavallée P. Two-way Optimal Stratification Using Dynamic Programming In: Proceedings of the Section on Survey Research Methods. Virginia: American Statistical Association; 1988.

[33] Dalenius T. Sampling in Sweden: contributions to the methods and theories of sample survey practice. Almqvist and Wiksell; 1957.

[34] Dalenius T, Hodges JL. The choice of stratification points. Scandinavian Actuarial Journal. 1957;1957(3-4):198–203. doi: 10.1080/03461238.1957.10405970

[35] Taga Y. On optimum stratification for the objective variable based on concomitant variables using prior information. Annals of the Institute of Statistical Mathematics. 1967;19(1):101–129. doi: 10.1007/BF02911690

[36] Serfling RJ. Approximately optimal stratification. Journal of the American Statistical Association. 1968;63(324):1298–1309. doi: 10.1080/01621459.1968.10480928

[37] Singh R, Sukhatme BV. Optimum stratification. Annals of the Institute of Statistical Mathematics. 1969;21(1):515–528. doi: 10.1007/BF02532275

[38] Singh R, Sukhatme BV. Optimum stratification in sampling with varying probabilities. Annals of the Institute of Statistical Mathematics. 1972;24(1):485–494. doi: 10.1007/BF02479777

[39] Singh R, Sukhatme BV. Optimum stratification with ratio and regression methods of estimation. Annals of the Institute of Statistical Mathematics. 1973;25(1):627–633. doi: 10.1007/BF02479404

[40] Singh R, Parkash D. Optimum stratification for equal allocation. Annals of the Institute of Statistical Mathematics. 1975;27(1):273–280. doi: 10.1007/BF02504646

[41] Mehta SK, Singh R, Kishore L. On optimum stratification for allocation proportional to strata totals. Journal of Indian Statistical Association. 1996;34:9–19.

[42] Rizvi SEH, Gupta JP, Bhargava M. Optimum stratification based on auxiliary variable for compromise allocation. Metron. 2002;60(3-4):201–215.

[43] Gupta RK, Singh R, Mahajan PK. Approximate optimum strata boundaries for ratio and regression estimators. Aligarh Journal of Statistics. 2005;25:49–55.

[44] Singh S. Advanced Sampling Theory With Applications: How Michael”” Selected”” Amy. vol. 2 Springer Science & Business Media; 2003.

[45] Thomsen I. A comparison of approximately optimal stratification given proportional allocation with other methods of stratification and allocation. Metrika. 1976;23(1):15–25. doi: 10.1007/BF01902846

[46] Hedlin D. On the stratification of highly skewed populations. Stockholm University. Mathematical Statistics; 1998.

[47] Neyman J. On the two different aspects of the representative method: the method of stratified sampling and the method of purposive selection. Journal of the Royal Statistical Society. 1934; p. 558–625. doi: 10.2307/2342192

[48] Bellman RE. Dynamic Programming. Princeton, N.J.: Princeton University Press; 1957.

[49] Taha HA. Operations Research: An Introduction. New Jersey: Pearson Education, Inc.; 2007.

[50] Cochran WG. Comparison of methods for determining stratum boundaries. Bulletin of the International Statistical Institute. 1961;38(2):345–358.

[51] Patten SB. A major depression prognosis calculator based on episode duration. Clinical Practice and Epidemiology in Mental Health. 2006;2(1):13 doi: 10.1186/1745-0179-2-13 16774672 10.1186/1745-0179-2-13 PMC1534018

[52] Wahed AS, Luong TM, Jeong J. A new generalization of Weibull distribution with application to a breast cancer data set. Statistics in medicine. 2009;28(16):2077–2094. doi: 10.1002/sim.3598 19424958 10.1002/sim.3598 PMC3057135

[53] Niu G, Singh S, Holland SW, Pecht M. Health monitoring of electronic products based on Mahalanobis distance and Weibull decision metrics. Microelectronics Reliability. 2011;51(2):279–284. doi: 10.1016/j.microrel.2010.09.009

[54] Wang H, Wang Z, Li X, Gong B, Feng L, Zhou Y. A robust approach based on Weibull distribution for clustering gene expression data. Algorithms for Molecular Biology. 2011;6(1):14 doi: 10.1186/1748-7188-6-14 21624141 10.1186/1748-7188-6-14 PMC3118357

[55] Hansen P, Jaumard B. Cluster analysis and mathematical programming. Mathematical programming. 1997;79(1-3):191–215. doi: 10.1007/BF02614317

[56] Baillargeon S, Rivest LP. The construction of stratified designs in R with the package stratification. Survey Methodology. 2011;37(1):53–65.

